# Binocular portraiture

**DOI:** 10.1177/20416695231165142

**Published:** 2023-04-16

**Authors:** Nicholas J. Wade

**Affiliations:** Psychology, University of Dundee, Dundee, UK

**Keywords:** anaglyphs, stereoscopic vision, binocular rivalry, portraits, photography, binocular art

## Abstract

Pictorial portraits are viewed with two eyes despite the fact that they are mostly monocular: they have been produced from a single viewpoint (either by painters or photographers). The differences between the images on each eye are a consequence of the separation between them rather than differences in two pictorial images. Viewing with two eyes detracts from the monocular cues to depth within the singular portrait because of information for the flatness of the pictorial surface. Binocular portraits, on the other hand, incorporate differences between two pictorial images producing perceptual effects that cannot be seen by a single eye alone. The differences can consist of small disparities that yield stereoscopic depth or large ones that produce binocular rivalry. Binocular portraits require viewing with a stereoscope, many varieties of which exist. Those shown here are anaglyphs which can be observed through red/cyan filters. They are not conventional stereoscopic portraits where the sitter is imaged from two slightly different locations. Rather, the binocular processes of cooperation (stereoscopic depth perception) and competition (binocular rivalry) are manipulated in the binocular portraits. The subjects shown in the anaglyphic portraits have been involved in the science and art of binocular vision.

## Introduction

The characteristics of picture perception have been investigated in the context of art and science (see Brooks, 2017; Erkelens, 2018). The vast majority of pictures we look at are essentially monocular: they appear much the same when viewed with one or two eyes. The differences between the images on each eye are a consequence of the separation between them rather than differences in two pictorial images: among the reasons for this are the histories of artistic representations of space and scientific investigations of spatial vision. Since the early 15th century pictorial art in general has been dominated by a monocular system for representing space—linear perspective. Throughout most of its history, investigations of binocular vision have been concerned with its singularity; that is, single vision with two eyes has been the principal topic. The situation might have been expected to change with the almost synchronous announcements of stereoscopy and photography (in the late 1830s) but this was not the case. While stereoscopic photographs of sitters were made, the apparent depth of facial features was incidental to that of the surrounding objects. The points above apply to stereoscopic portrayals; even less interest has been given to those involving binocular rivalry despite its longer history than stereopsis. A further force that has acted against binocular portraiture is the need for some viewing device to combine the paired pictures.

**Figure 1.**
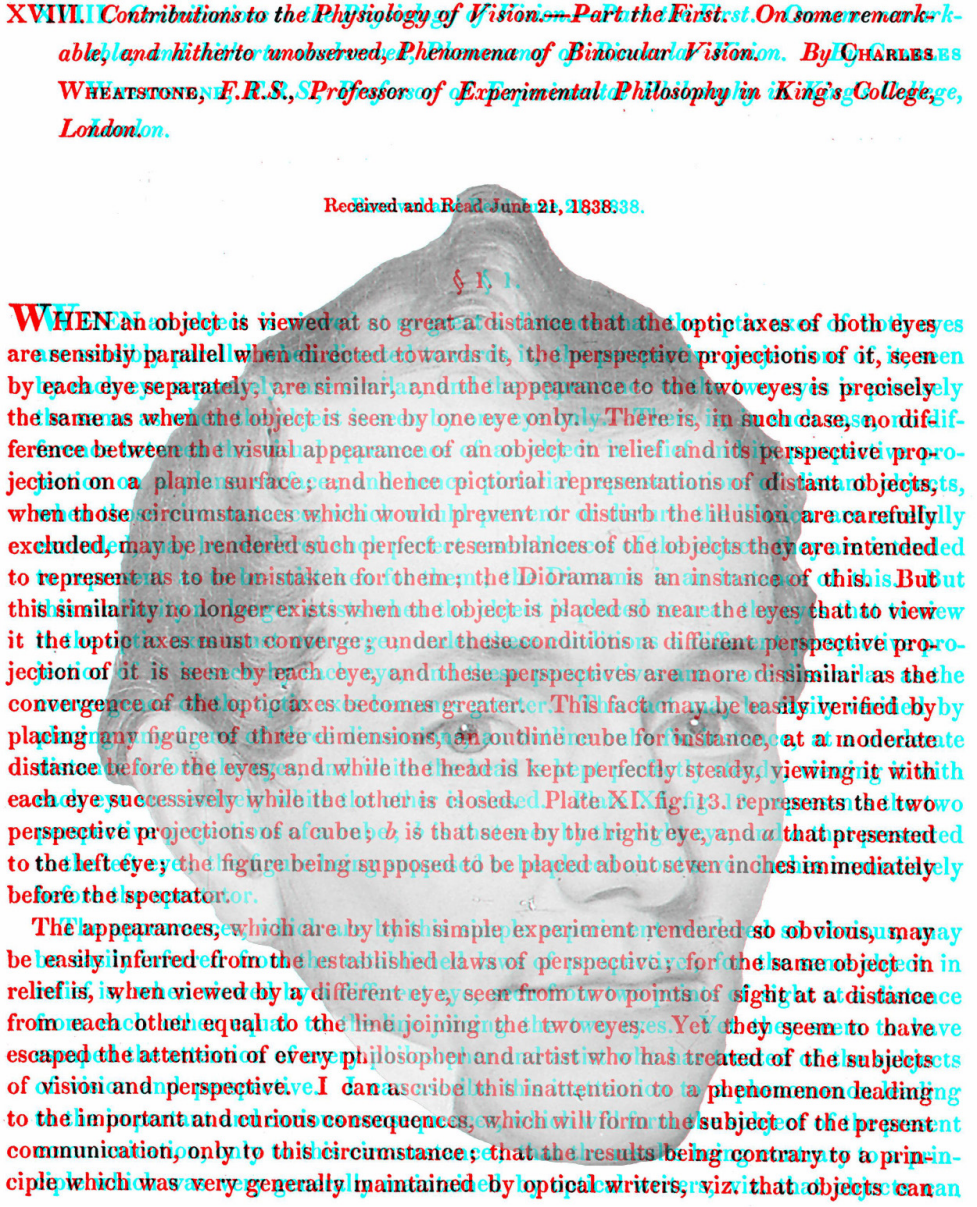
*Charles Wheatstone and his announcement of the stereoscope* by Nicholas Wade. The monocular images each contain the same partially transparent portrait of Wheatstone so that the text continues through it. The location of the portrait with respect to the text is shifted laterally so that when the portraits are aligned the text displays disparities. This can be seen by viewing the two monocular images separately. When viewed with the red/cyan filters in front of the eyes the partially transparent portrait appears either closer than the text or more distant.

My intention is to present novel ways in which portraits can be displayed binocularly, like that in Figure 1. The portrait is derived from a painting of a young Charles Wheatstone who had the first stereoscopes made in 1832 and he announced his invention to the public in 1838. As with all the illustrations that follow, Figure 1 is an anaglyph requiring red/cyan viewers to see the depths and/or rivalries visible with them. Anaglyphs are simple stereoscopes that have an advantage over mirror and prism stereoscopes in that there are four possible outcomes from viewing them rather than three: with each eye alone to see the monocular images, with both eyes to see them in stereoscopic depth or rivalry, or without the red/cyan glasses where they can have an appeal independent of the binocularity they encompass. Reflecting and refracting (optical) stereoscopes do not offer this last possibility. Although anaglyphs do not separate the patterns to each eye as fully and equally as optical stereoscopes, they do have the advantage that the superimposition of red and cyan images creates a third design that can have an allure of its own. The disadvantage of anaglyphs is that the color characteristics of the monocular images are not retained. Some examples of anaglyphic portraits can be seen in Wade (2019, 2021a, 2021b, 2021c, 2021d, 2023). The subjects shown in the anaglyphic portraits illustrated in this article have been involved in the science and art of binocular vision.

Conventional stereoscopic portraits rarely reflect the depth of the facial features. Rather it is the depth of the sitter with respect to the foreground and/or background objects that is apparent. This aspect of portraiture has been manipulated in Figure 1. That is, the disparities are confined to the background rather than the person portrayed. I am not aware of the intentional use of this technique in stereoscopic portraiture. The seemingly transparent portrait of Wheatstone can be seen in depth relative to the text surrounding it and the sign of depth will reverse with reversal of the color filters. That is, the text appears either nearer or farther than the portrait which is itself not stereoscopic. It looks as if the transparent features of the face are seen behind the text or they hover in front of it. The effects might take some seconds for the depths of the portrait to articulate in this way. The distant face occurs with the red/left eye and cyan/right eye arrangement, and the hovering face with the reverse. The text is the opening page of Wheatstone's (1838) paper to the Royal Society describing his invention of the stereoscope and the experiments he conducted with it.

**Figure 2.**
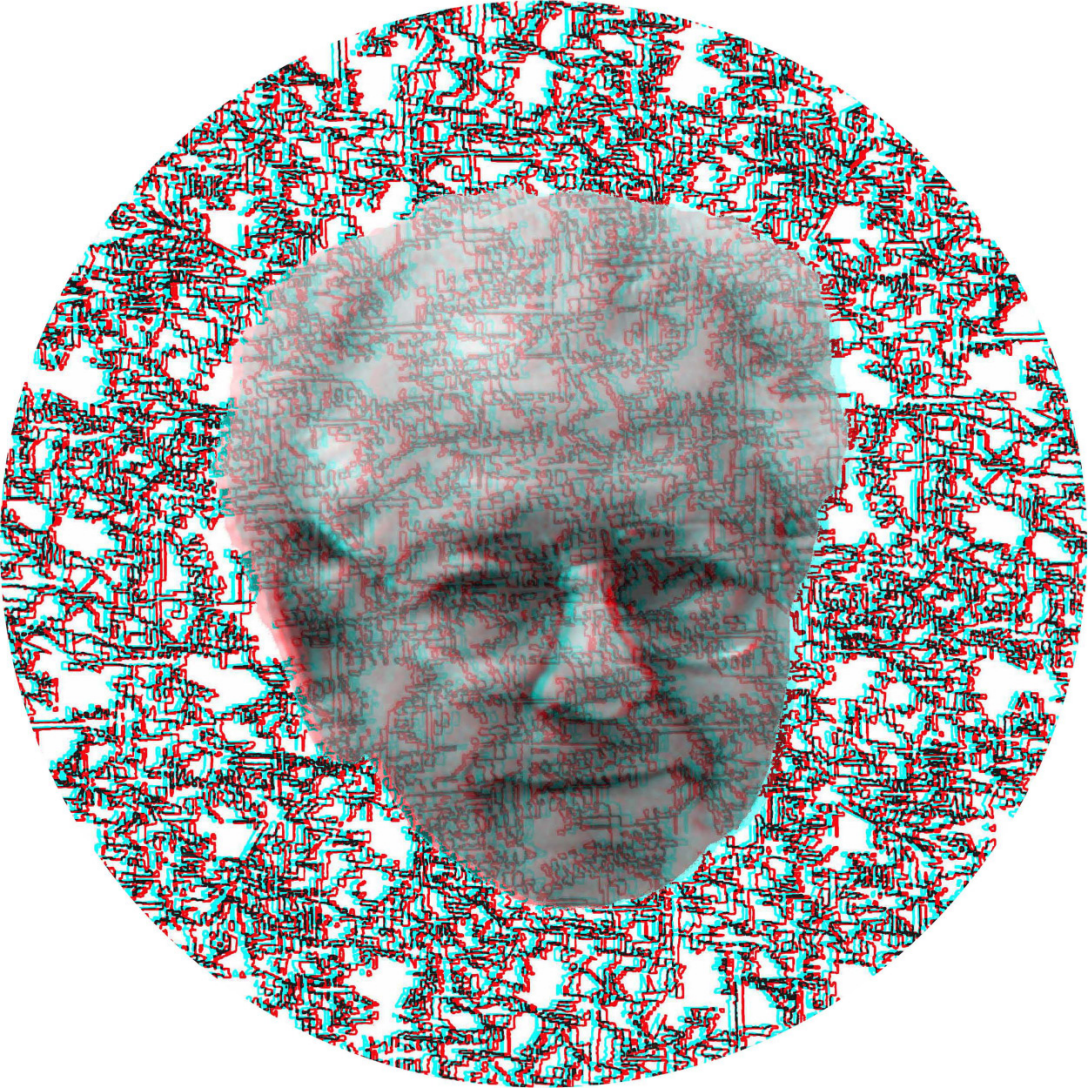
*Béla Julesz in double depth* by Nicholas Wade. The portrait of Julesz itself appears in depth and in front of the background pattern with the red/left eye and cyan/right eye combination. Reversing the color filters results in the whole face appearing more distant than the background but the features of the face do not reverse.

When there are apparent depths of facial features in conventional stereoscopic portraits they do not reverse with reversal of the filters, as can be seen in Figure 2. The portrait of Béla Julesz is embedded in a silhouette of his face which does reverse in depth, unlike the facial features that are carried by a random dot pattern. The portrait is based on a stereoscopic photograph taken by Thomas Papathomas and Kazunori Morikawa (see Papathomas et al., 2019) and the background was derived from a random dot pattern.

The reversals in apparent depth of monocular images in optical stereoscopes were discussed by Wheatstone (1838) and such “conversions of relief” as they were called were elaborated upon in his second memoir (Wheatstone, 1852). Simple geometrical figures reverse readily but figures involving overlap or superimposition were difficult to see in reversed depth. Brewster (1856) gave a graphic description of viewing a stereoscopic portrait with the monocular images reversed: “Although the nose of the human face should retire behind the ears yet no such effect is produced, as all the features of the face are connected with each other, but if the nose and ears have been represented separately in the position which they occupy in the human head, the nearer features would have retired behind the more remote ones, like the separate articles on a table” (p. 210). Wheatstone (1852) made an optical device for reversing disparities when viewing solid objects which he called a pseudoscope. Several variations on his prism pseudoscope were subsequently produced, often involving mirrors (see Jastrow, 1900). Indeed, Jastrow remarked that “it is difficult to see pseudoscopically the human face” (p. 49). These observations were confirmed by Wallin (1905) in his extensive survey of reverse perspective. The failure of the reversed disparities to reverse the appearance of a face is similar to the hollow mask illusion, described by Brewster (1826). When discussing conversions of relief in shadows cast in intaglios he noted that “We have succeeded in carrying out this deception so far, as to be able, by the eye alone, to raise a complete hollow mask of a human face into a projecting head” (p. 108).

With the onset of computer graphics it became possible to generate designs in which depth only emerges when the component patterns are viewed with two eyes (Julesz, 1960, 1971), although there were graphical precursors of this (see Blundell, 2011; Howard & Rogers, 1995; Wade, 2021b). For example, Kompaneysky (1939) made paired patterns of white dots on a black background which carried the face of Venus when combined in a stereoscope. Research on stereoscopic depth perception was transformed by the introduction of random dot stereograms and its influences rippled through pictorial art, too. Those devised by Julesz were pairs of matrices of squares in which the contents of each cell were randomly assigned as black or white; displacing a region in one display and combining it with an unmodified pattern in a stereoscope resulted in that region appearing in depth. Using such patterns it was possible to examine stereopsis without monocular cues for the appearance of depth.

**Figure 3.**
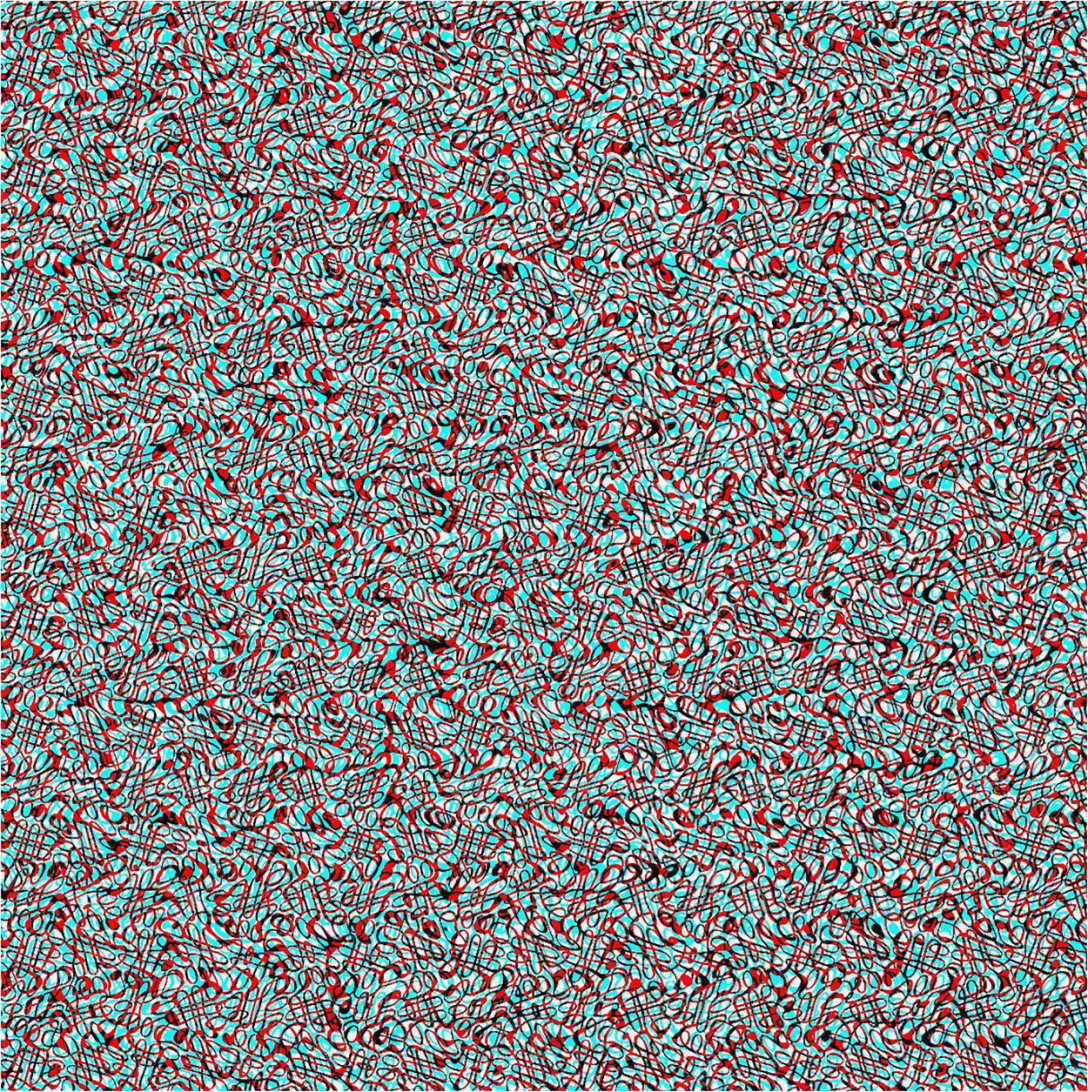
*Profile in depth* by Nicholas Wade. A right-facing profile (from the forehead to the neck) in the center of the pattern is defined by differences in depth so that it appears more distant with red/left eye and cyan/right eye and closer with the reverse combination.

**Figure 4.**
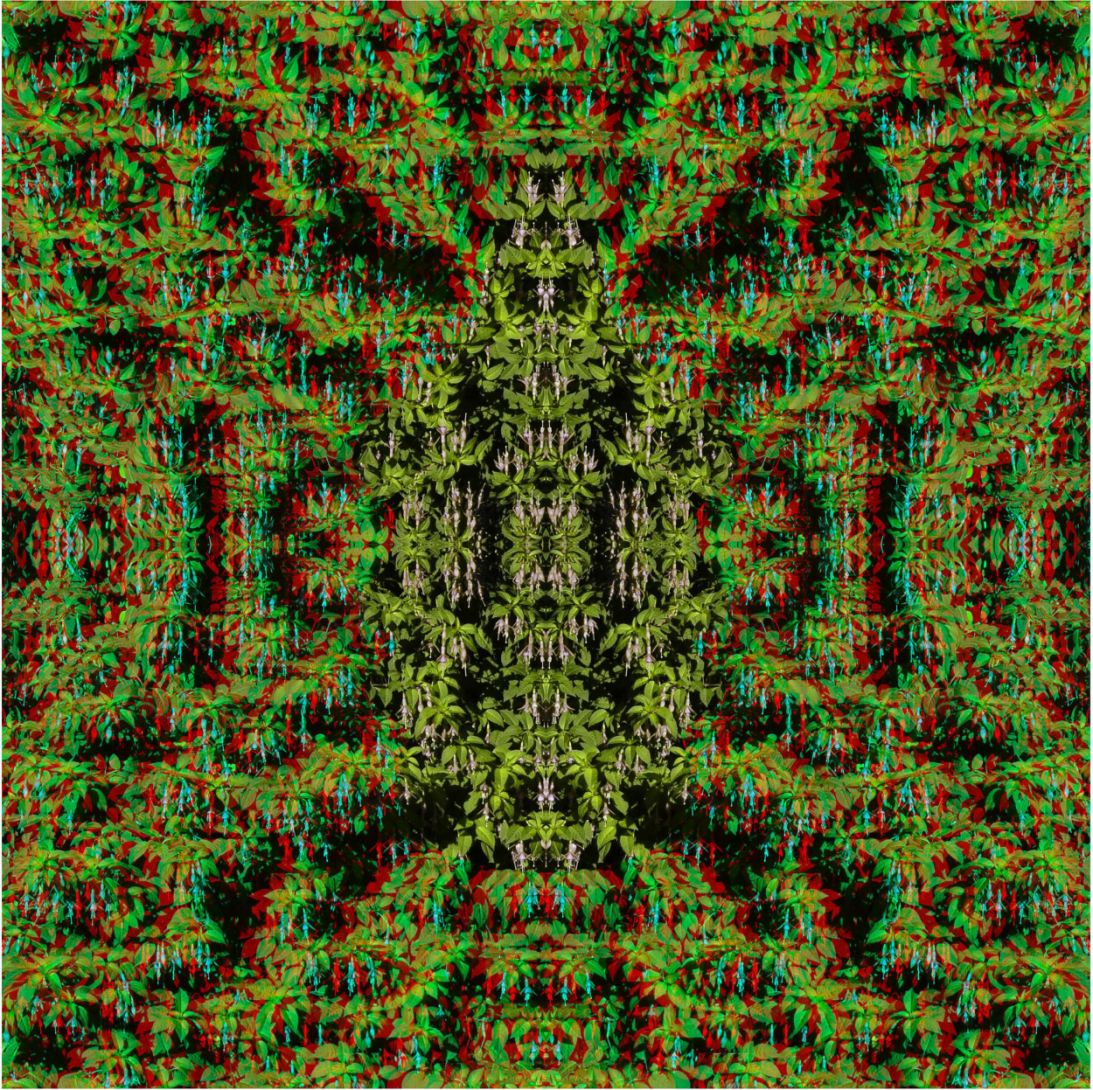
*Profiles in depth* by Nicholas Wade. The same profile faces left and right with an overlapping region in the center corresponding to that between the lower forehead and the chin. The overlapping region can be seen in a depth plane between those of the remaining parts of the heads.

Computer-generated random dot patterns do not have a great deal of visual allure in themselves, and more complex “carrier patterns” for stereoscopic pictures can be enlisted. Those I have made are derived from either graphic designs or photographs of natural patterns. They are not only more complex than random dot patterns but they can also have an appeal independently of the depth they contain. An example of the former is shown in Figure 3 and of the latter in Figure 4; the starting points for the illustrations were either graphic designs or natural textures, respectively. They were scanned or photographed and digitally modified to produce the carrier patterns which can be paired and combined to make the anaglyphs using StereoPhotomaker (https://stereo.jpn.org/eng/stphmkr/). Stereoscopic depth can be induced by disparities over the whole pattern surface or of contents within it or both and rivalry can be produced with an inclusion, like a portrait, in just one of the carrier patterns. The components of Figure 3 were initially regular arrays of 0 s which were distorted and superimposed to make the texture that can be seen by each eye alone. In one member of the pair a region corresponding to a profile was displaced and combined with the unchanged pattern. The differences between the two components cannot be detected monocularly. When viewed with red/cyan glasses the profile will emerge in depth; reversing the color filters will reverse the apparent depth.

The carrier pattern in Figure 4 was derived from a photograph of fuschia flowers and the depths within it when combined are more complex and may take longer to emerge. Two profiles, facing in opposite directions, are superimposed and their superimposition is in an intermediate depth plane to the remainders of the heads.

**Figure 5.**
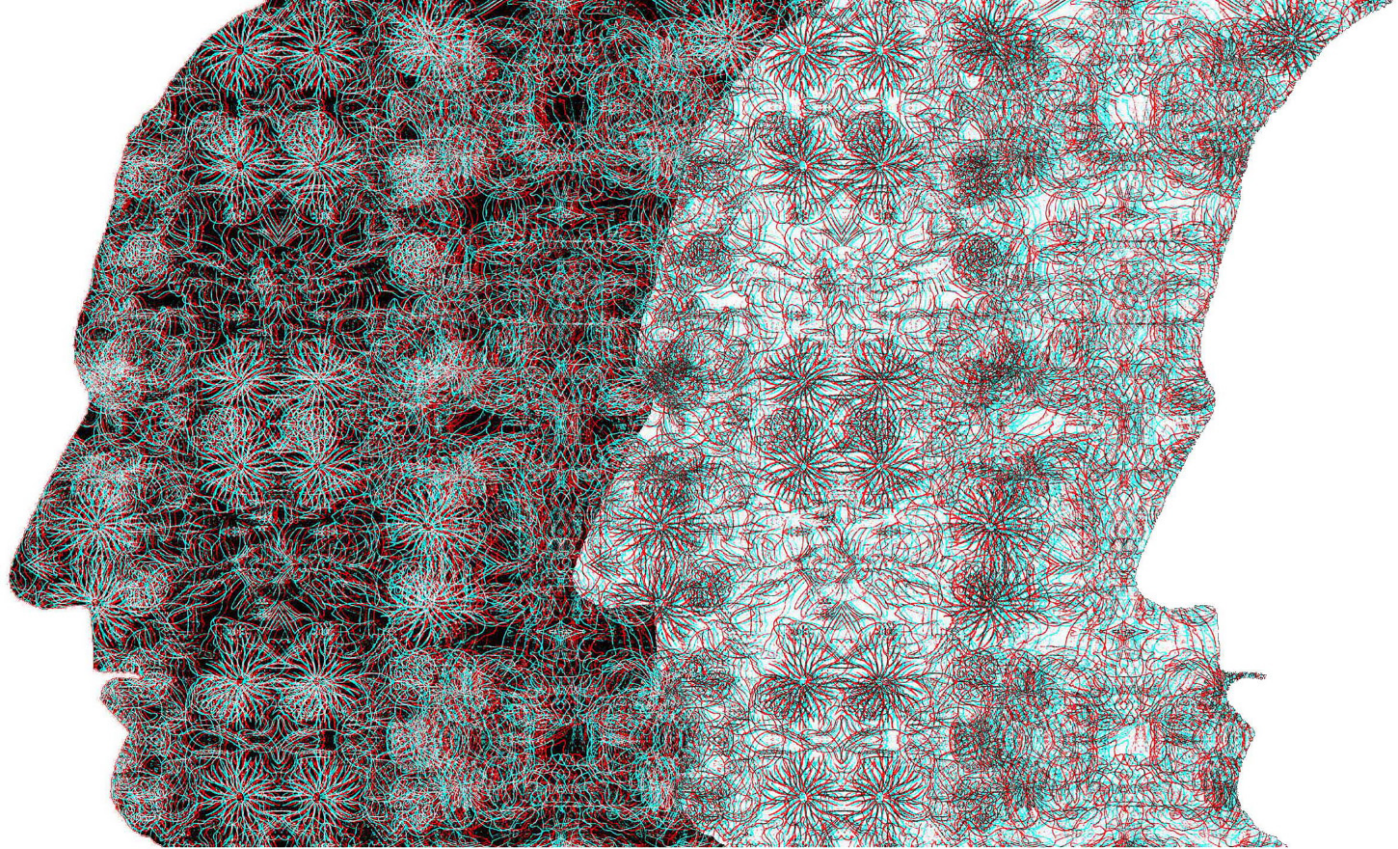
*Tom Wedgwood in profiles* by Nicholas Wade. The three clearly visible profiles have two equivalent profiles between them defined by disparity differences and thereby depth.

Profiles provide more stereoscopic opportunities than do full face portraits as is shown in Figure 5. The profile is of Tom Wedgwood, who was “The first person to attempt to record the camera image by means of the action of light” (Newhall, 1982, p. 13). Tom was a son of the potter Josiah Wedgwood and he made images from focused sunlight, but he could not fix them (see Wade, 2005). His profile is repeated five times, carried by a pattern derived from a painted Wedgwood plate. Thus it is possible to marry a pattern in depth with the portrait that it carries.

**Figure 6.**
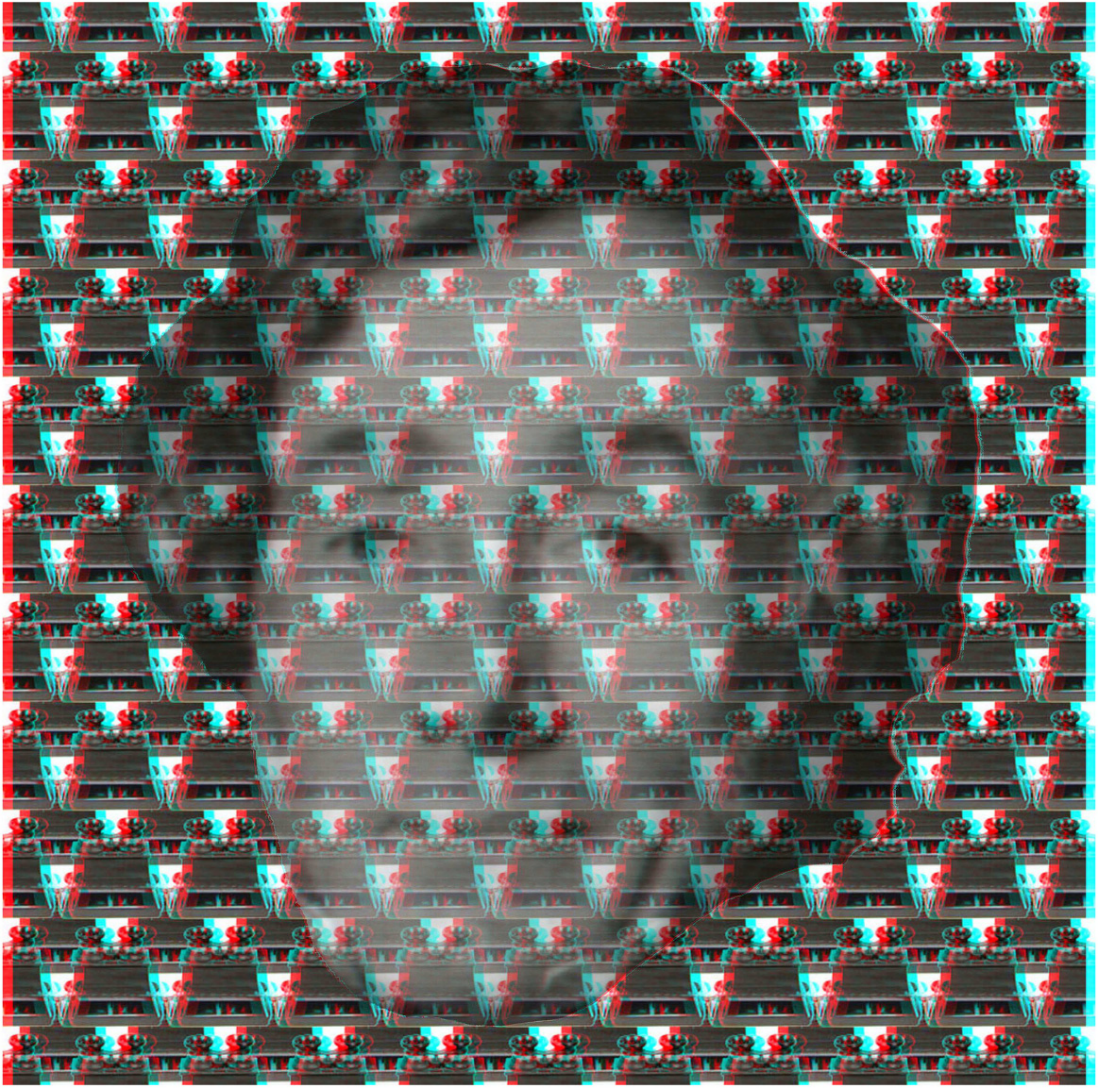
*Stereoscopic Brewster and his stereoscope* by Nicholas Wade. The technique here employed is essentially similar to that for Wheatstone in Figure 1. Two equivalent, partially transparent portraits of Brewster were differentially displaced relative to the pattern of stereoscopes and then combined to align the portraits with consequent disparities in the arrayed stereoscopes. When viewed with the red/left eye and cyan/right eye combination it is as though the portrait is seen through the transparent stereoscopes whereas the reverse arrangement results in the appearance of the stereoscopes through a partially transparent portrait.

Brewster's lenticular (refracting) stereoscope was announced in 1849 and it proved more popular than Wheatstone's earlier mirror (reflecting) stereoscope largely because of its portability and convenience for mounting paired photographs. A version of Brewster's stereoscope carries his paired portrait in Figure 6. As is the case with Wheatstone's portrait in Figure 1, Brewster's face is seen either beyond the array of stereoscopes or appears to hover in front of it.

## Stereoscopic Portraits

**Figure 7.**
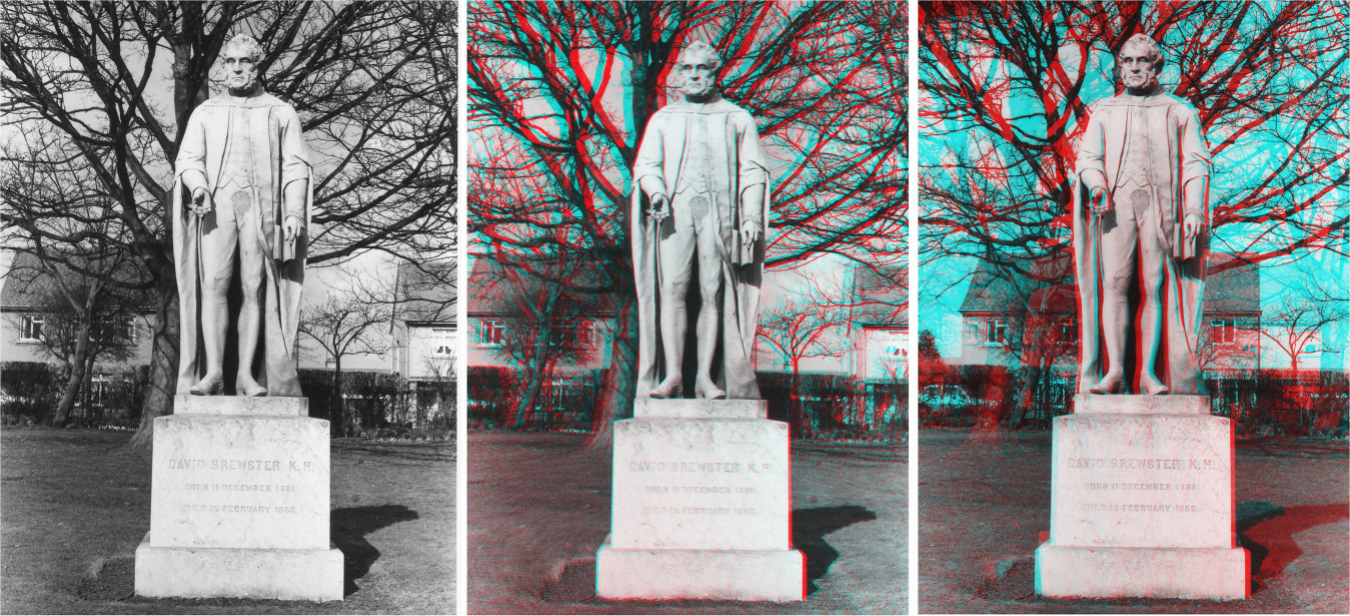
*David Brewster’s statue* by Nicholas Wade. Monocular (left) and stereoscopic photographs of Brewster's statue. The separation between the cameras was about 6.5 cm for the central stereoscopic photograph and about 100 cm for the one on the right.

Stereoscopic photographs can be taken simultaneously with a binocular camera or successively with a single-lensed camera. The first stereo-portraits were taken with a single camera moved laterally between exposures because that was the easiest method then available. Binocular cameras were not devised until the 1850s (see Pellerin and May, 2021; Wing, 1996). Statues lent themselves to sequential exposures and in 1841 the inventor of the stereoscope (Charles Wheatstone) asked the inventor of positive/negative photography (William Henry Fox Talbot) to take stereoscopic photographs of a statue at Laycock Abbey. Unfortunately, the separation between the two exposures was excessive but it raised the important question of the suitable separations for the left and right eye images. Later, Brewster (1856) maintained that the separation (as in his binocular camera) should correspond to the distance between the eyes. Wheatstone (1852) was more pragmatic and provided a table of convergence angles for objects at different distances. An example of this difference in approach can be seen with the stereo-photographs of Brewster's statue which stands outside King's Buildings at Edinburgh University (Figure 7). The stereoscopic photographs were taken with a single camera. The distance from the statue was about 6 m and the camera separation for the center pair was approximately 6.5 cm and for the right pair it was about 100 cm. The statue by William Brodie was erected in 1871 and unseen from the camera angles adopted a small stereoscope protrudes beneath Brewster's robes. There are few differences in the perception of facial features in the three photographs despite the large camera separation in the rightmost one. Conventional stereoscopic photographs, like these, should be viewed with red filter in front of the left eye and the cyan filter in front of the right eye.

**Figure 8.**
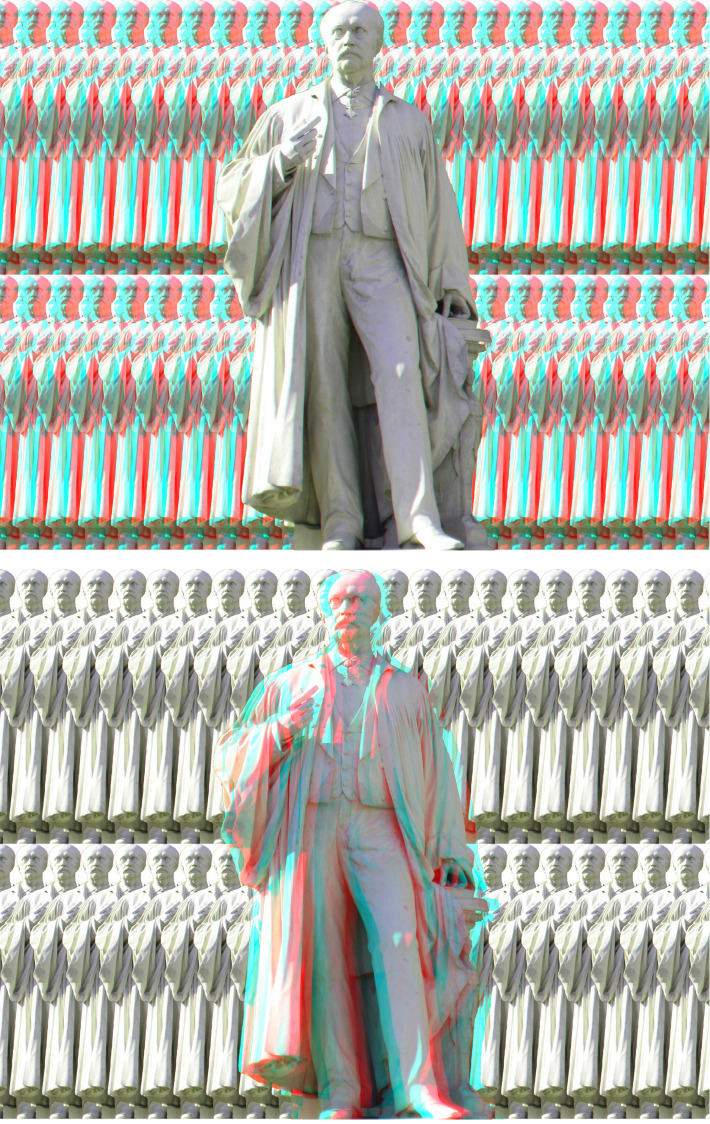
*Hermann Helmholtz in depths* by Nicholas Wade. In the upper figure the disparities are confined to the background, whereas the lower one is more like a conventional stereoscopic photograph. However, in the latter case it is the same photograph of the statue that is laterally displaced with respect to the background.

Statues can appear solid even though they have been photographed by a single-lensed camera. This can be seen in the statue of Helmholtz (Figure 8) which stands at the entrance to the Humboldt University in Berlin. The statue appears in depth both when the disparities are in the background (comprised of graphical multiplications of the same statue) alone or in the statue alone. The depth is marked with a red/left eye and cyan/right eye arrangement but does not reverse with the opposite combination due mainly to the occlusion common to both. However, the depth is less compelling in the latter case.

**Figure 9.**
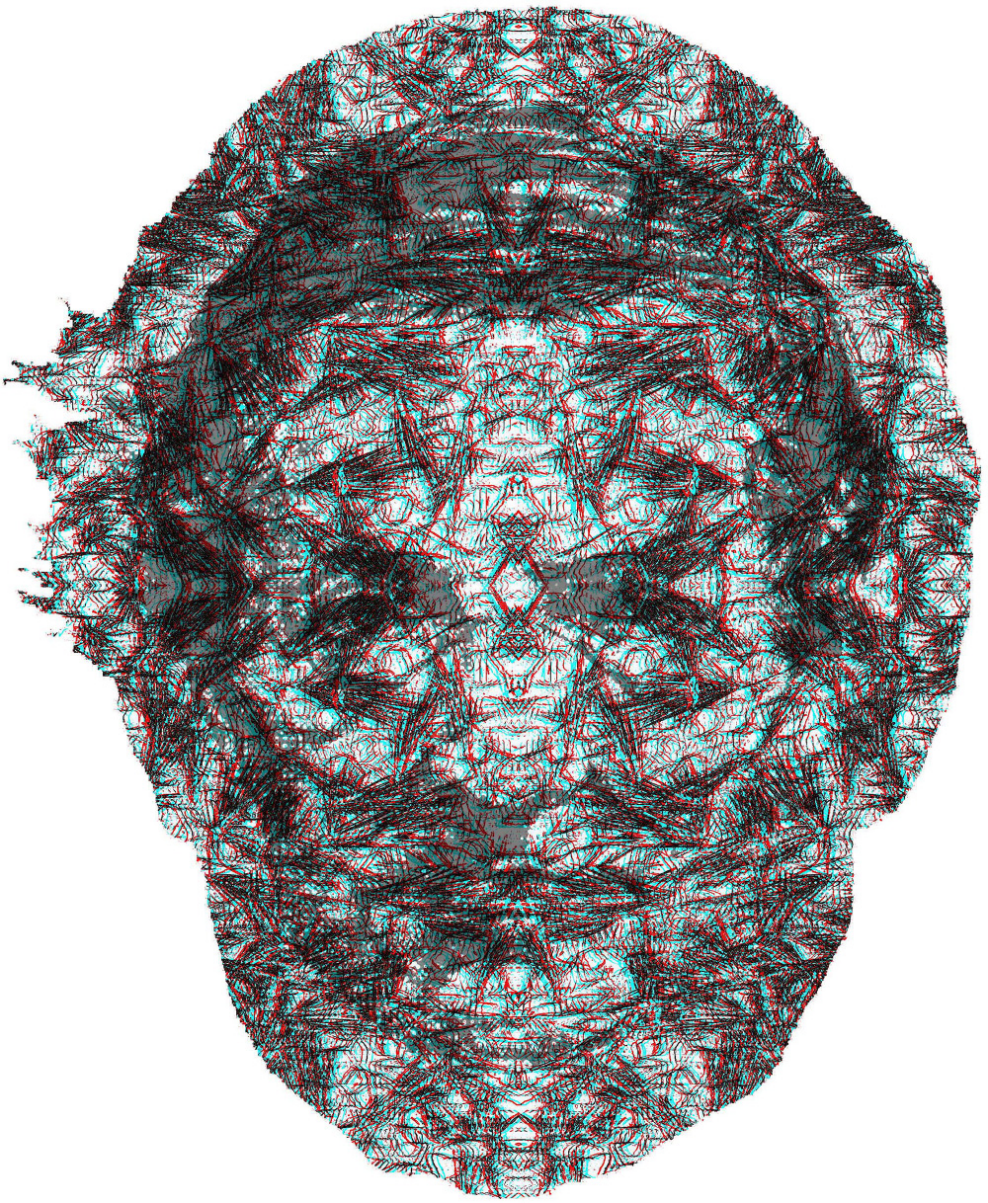
*Charles Wheatstone in depth* by Nicholas Wade. A silhouette of Wheatstone can be seen in depth with respect to a larger background the border of which is the same silhouette. The partially transparent full face portrait of Wheatstone was added to the anaglyph so that it is visible by either eye. The apparent depth is defined from the inner silhouette rather than the portrait of Wheatstone.

 Figure 9 is not a stereoscopic portrait in the conventional sense; the face of Wheatstone appears in one depth plane (either nearer or farther than the background) but does not contain any stereoscopic features itself. The original portrait is detail from an engraving and the pattern carrying his facial features was derived from graphically manipulated photographs of wheat and stones; the whole figure is framed within a silhouette of Wheatstone's portrait.

**Figure 10.**
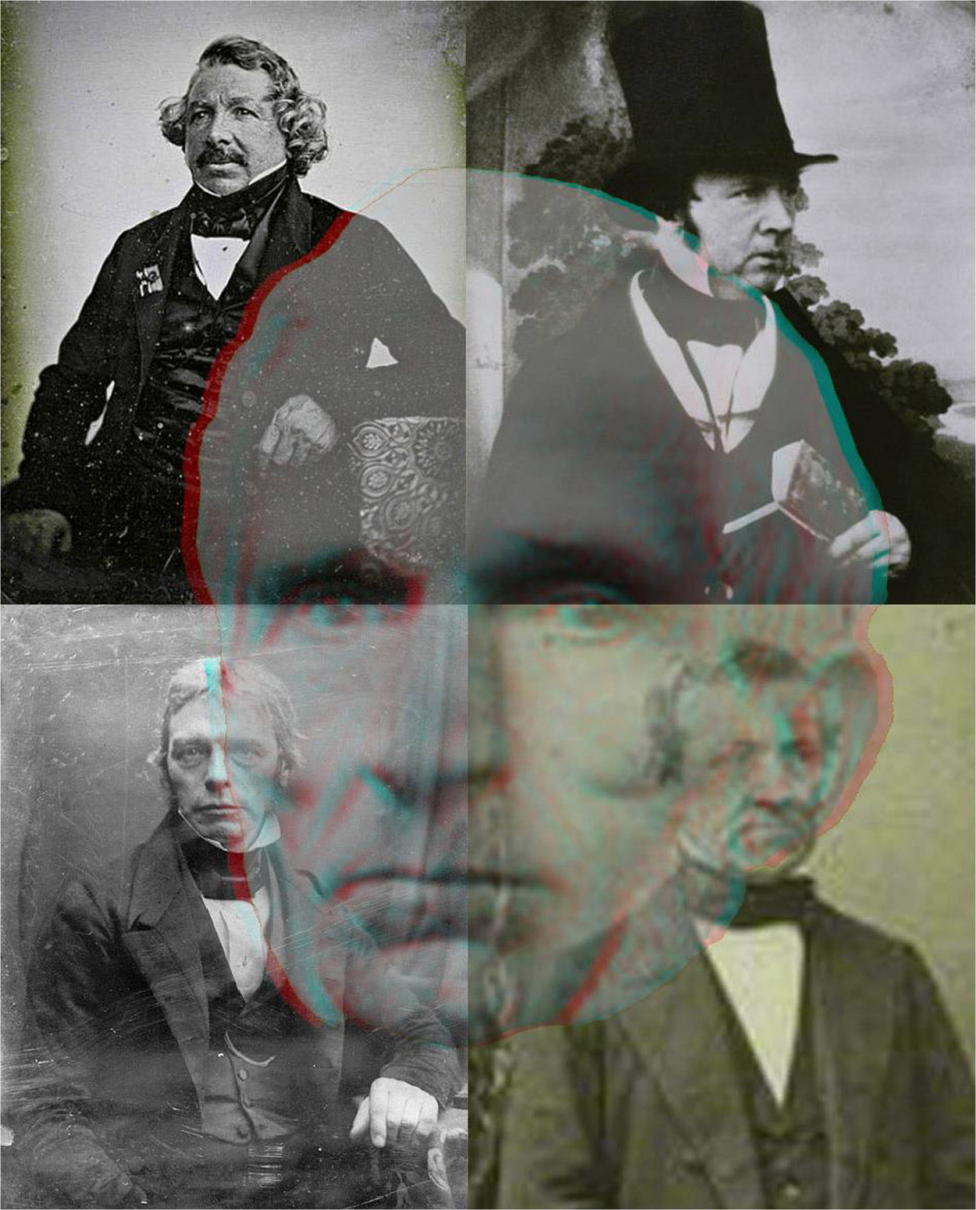
*Antoine Claudet in depth* by Nicholas Wade. Claudet can be seen in transparent depth combined with photographic portraits he took of Daguerre, Talbot, Wheatstone, and Faraday (clockwise from the top left).

A conventional stereoscopic daguerreotype of the Wheatstone family was taken by Antoine Claudet in 1854 (see Pellerin & May, 2021). Claudet was born in Lyon and moved to London in 1829. He opened the first daguerreotype studio in London and specialized in portraiture. He wrote extensively about photography and made one of the first devices to combine stereoscopic depth with apparent motion (see Wade, 2012). Claudet (Figure 10) was recognized as a scientist as well as a photographer (Gill, 1967). He advocated Wheatstone's procedures for taking stereoscopic photographs of objects: “the binocular angle of stereoscopic pictures must be in proportion to the ultimate size of the pictures on the retinas, larger than the natural angle when the images are magnified, and smaller when they are diminished” (Claudet, 1860, p. 22). He had earlier made an instrument called a stereoscopometer which calculated the angle required to take stereoscopic photographs of objects or groups. Unlike Figures 1 and 6, the disparities in Figure 10 are in the portrait rather than the background photographs.

**Figure 11.**
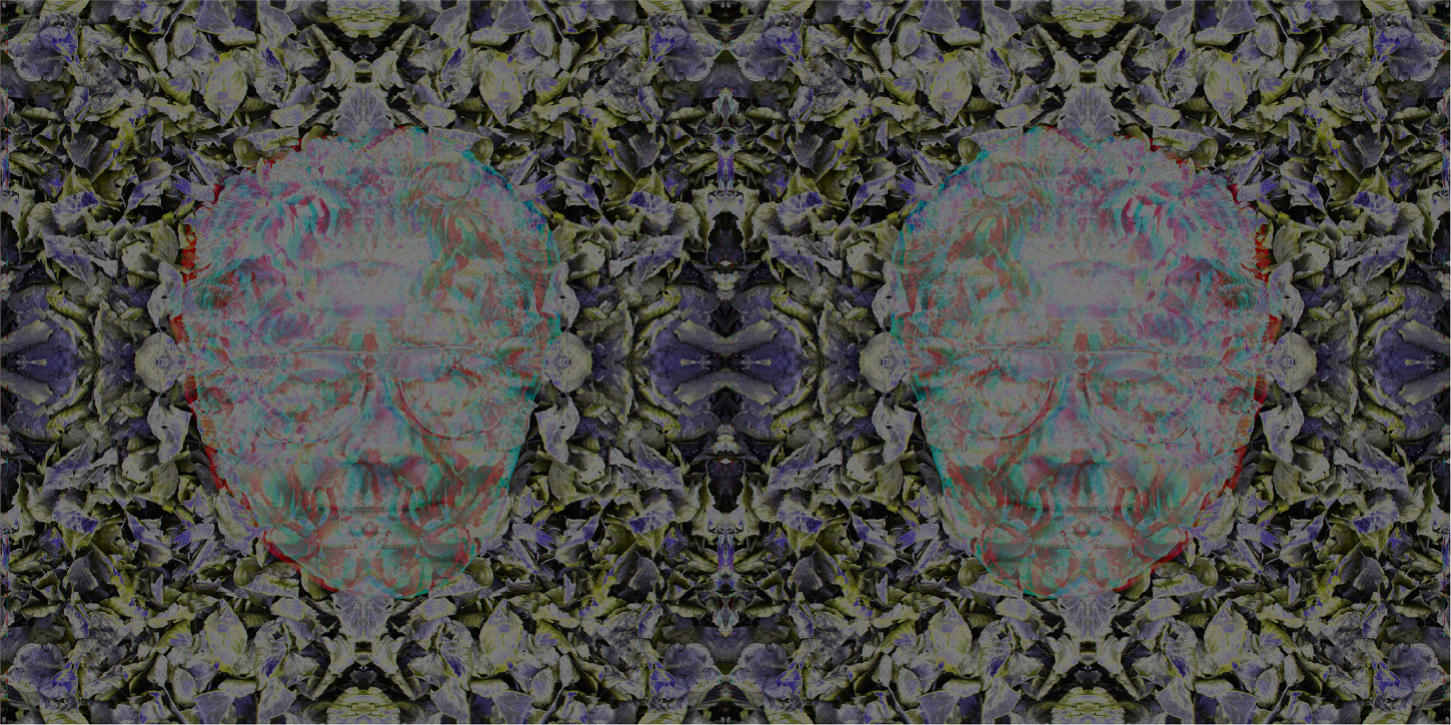
*Béla Julesz in leafy depths* by Nicholas Wade. A partially transparent portrait of Julesz was displaced relative to the leaf pattern and combined to make an anaglyph which was solarized. The solarized anaglyph was joined to its left–right reversal to create the final, symmetrical pairing.

Wheatstone (1852) championed the union between camera and stereoscope because he appreciated that consistent, small disparities between two pictures would be difficult to achieve otherwise. However, he was acutely aware that even simple line drawings for the stereoscope would still have monocular indications of depth. He could see no way of overcoming this and his dream was realized by Julesz over a century later. As noted above, the random dot patterns devised by Julesz do not have much artistic appeal and examples of more complex carrier patterns have been shown. An additional advantage of the latter is that a wider range of graphical techniques can be employed in creating them. The face of Julesz can be seen twice in stereoscopic depths in Figure 11. The carrier pattern is based on a photograph of fallen autumn leaves and the combined stereoscopic image was solarized to yield the darker and softer colors in the final stereogram. The flat faces reverse in depth with reversal of the color filters. Note how the nearer face appears to be smaller than the more distant one and this difference in apparent sizes reverses when the anaglyphic glasses are reversed. Wheatstone (1838) drew attention the apparent sizes changes when the monocular images are reversed—what he called the converse figure: “Those points which are nearest the observer in the proper figure are the most remote from him in the converse figure, and *vice versâ*, so that the figure is, as it were, inverted; but it not an exact inversion, for the near parts of the converse figure appear smaller, and the remote parts larger than the same parts before inversion. Hence the drawings which, properly placed, occasion a cube to be perceived, when changed in the manner described, represent the frustrum of a square pyramid with its base remote from the eye” (p. 377).

## Portraits in Binocular Rivalry

Binocular portraits are not restricted to stereoscopic depth perception, but they can involve it. Binocular rivalry offers far more potential than stereopsis for creating binocular portraits. They can reflect the same person in contrasting postures, at different ages or carried by appropriate graphical or textual motifs. The two monocular components need not be of the same person so that a wide variety of possibilities can be entertained (see Wade, 2021d).

Historical aspects of investigations of binocular vision can be presented by combining portraits and some suitable motifs reflecting their contribution. The fluctuating visibility when radically different patterns are presented to each eye was described by Porta (1593): “Place a partition between the eyes, to divide one from the other, and place a book before the right eye, and read; if another book is placed before the left eye, not only can it not be read, but the pages cannot even be seen, unless the visual virtue is withdrawn from the right eye and changed to the left” (pp. 142–143). Porta was addressing the problem of seeing the world as single despite two views of it and he provided a simple solution—only one eye is used at one time. The alternative interpretation of binocular single vision is that the two images are fused or combined in some way.

**Figure 12.**
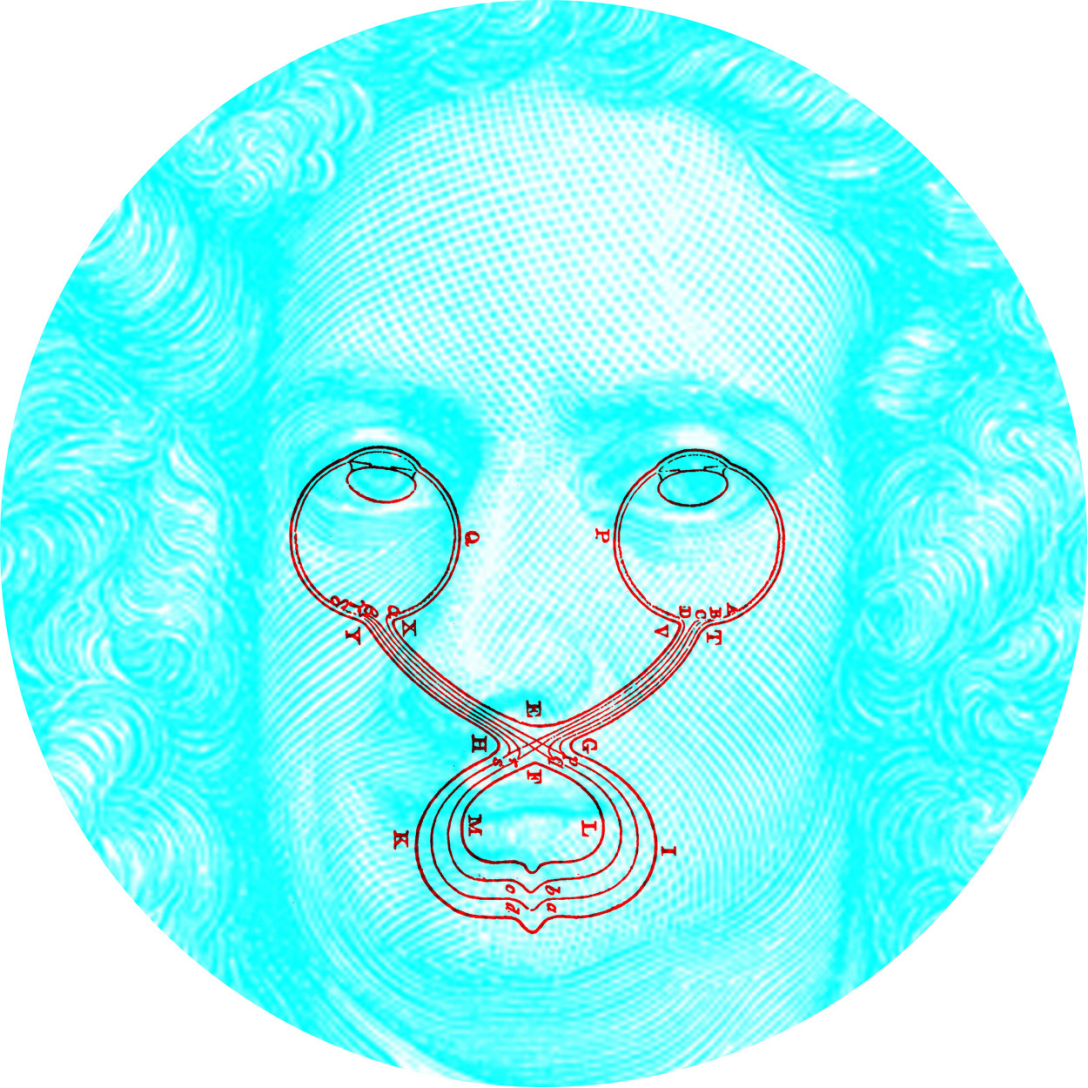
*Isaac Newton's visual pathways* by Nicholas Wade. Both the diagram of the visual pathways and the engraved frontispiece portrait of Newton are taken from Brewster (1855). The fibers from each eye are shown to partially decussate at the chiasm.

In his notebooks, Newton not only mused about binocular contour rivalry but he also speculated on its anatomical basis: “Why, though one thing may appear in two places by distorting the eyes, yet two things cannot appear in one place. If the picture of one thing fall upon A, and another upon α [its corresponding point in the other eye], they may both proceed to *p* [in the optic chiasm], but no farther; they cannot both be carried on the same pipes *pa* into the brain; that which is strongest or most helped by phantasy will there prevail, and blot out the other” (Harris, 1775, p. 110, parentheses added). The illustration to which this applies is shown in Figure 12, together with a portrait of Newton. The drawing of the pathways was much less precise in Newton's notebook but it was redrawn by Harris (1775) and by Brewster (1855) in his biography of Newton (from which the portrait was derived). Each component can be seen separately by closing one eye at a time. Unfortunately, Newton was wrong in his anatomy—the two “pipes” (nerve fibers) in the optic nerve do not unite in the optic chiasm but remain separate (see Howard & Rogers, 1995; Wade, 1998). Nonetheless, he was seeking to examine binocular vision within the framework of his science and to give an interpretation of visual phenomena in mechanistic terms.

**Figure 13.**
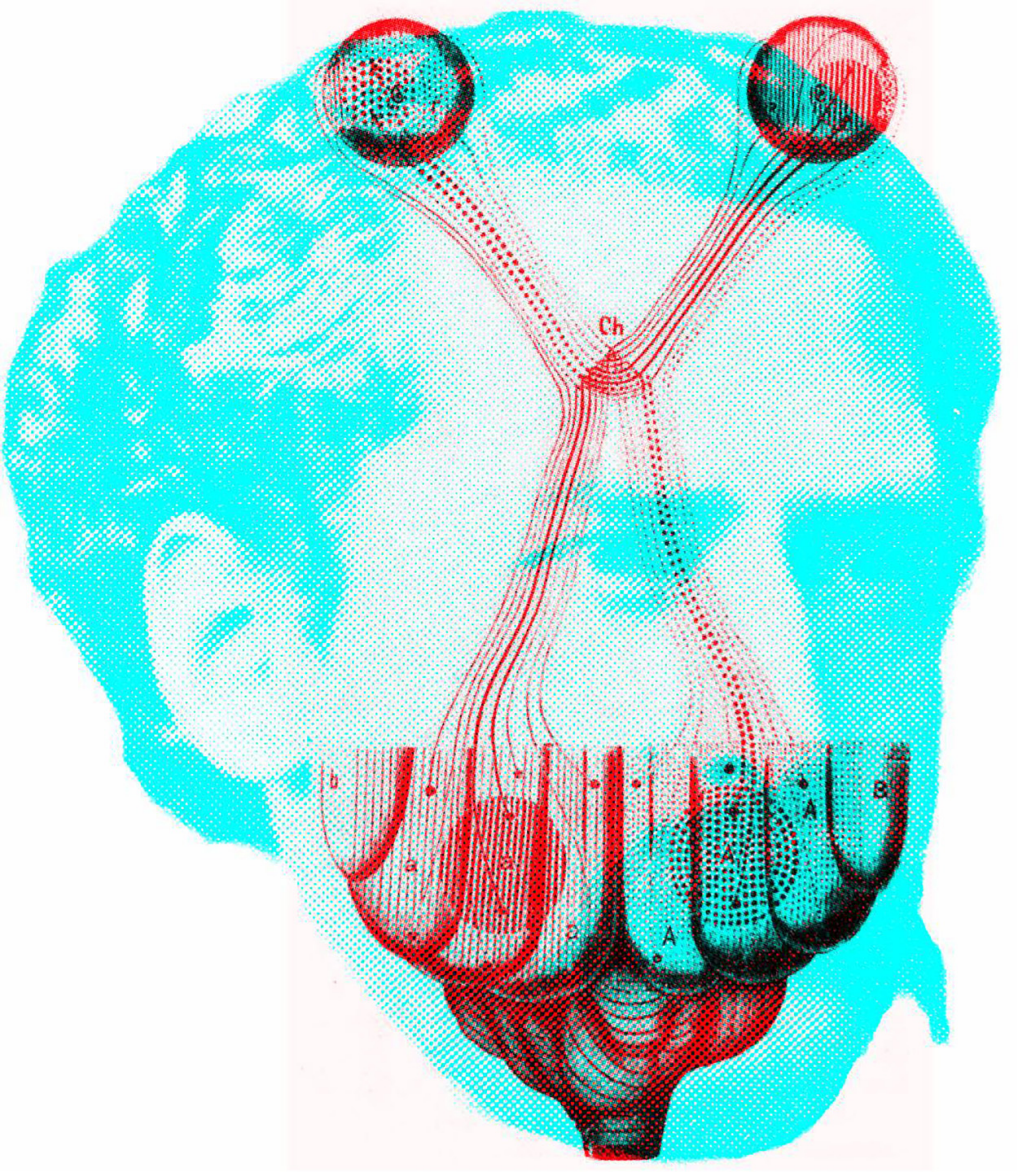
The visual pathways of Hermann Munk by Nicholas Wade.

There was much debate and controversy surrounding partial decussation at the optic chiasm. It was resolved by Munk (1879) who pursued the pathways from the eyes to the brain. Disputes about partial decussation at the optic chiasm had raged for a century and a half but Munk's painstaking dissections and beautiful illustrations established the routes taken from eyes to brain (Figure 13). Munk went on to examine the visual cortical functions in greater detail. He found that cortical lesions in the visual areas produced two types of blindness which he called psychic and cortical. Psychic blindness resulted in the experimental animals (dogs and monkeys) behaving appropriately to objects (e.g., by avoiding collision with them) but showing no evidence of recognizing what they were. Cortical blindness, on the other hand, reflected total absence of vision, and it typically followed complete removal of the primary visual cortex.

**Figure 14.**
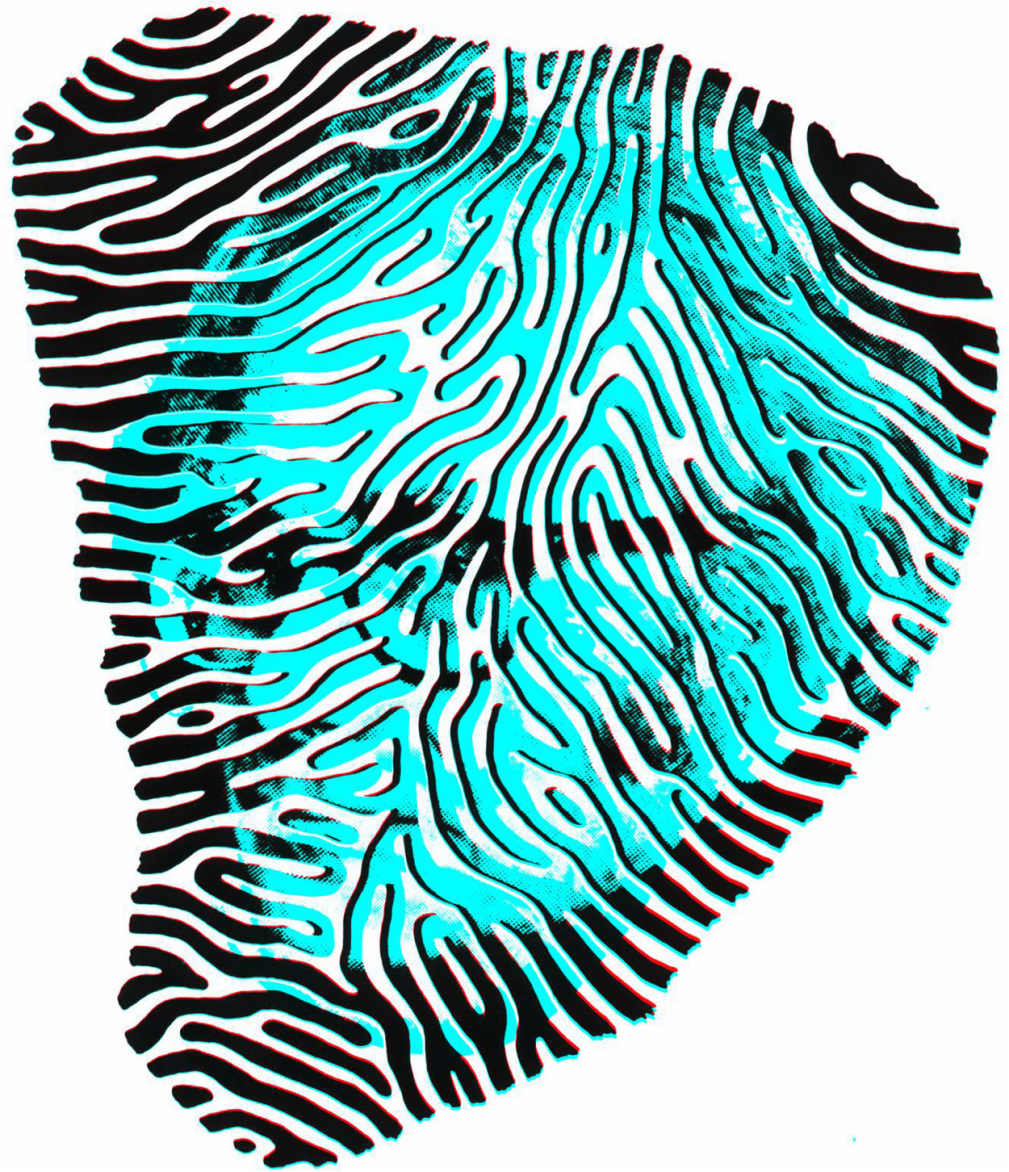
*The ocular dominances of David Hubel and Torsten Wiesel* by Nicholas Wade. Hubel can be seen through the cyan filter and Wiesel through the red.

The binocular rivalry seen in the last two illustrations is restricted to local areas of the portraits whereas it can involve larger areas of common correspondence, as is the case for Figure 14. Recordings from single cells in primate visual cortex extended understanding of visual functioning considerably. Feature detectors were isolated and their architectures within areas of the visual cortex were mapped. One of the aspects of cell specificity was that binocular cells could be more strongly influenced by one eye or the other. The portraits of Hubel and Wiesel (Figure 14) are embedded in an illustration of the ocular dominance columns in macaque monkey visual cortex that they mapped (Hubel and Wiesel, 1977).

**Figure 15.**
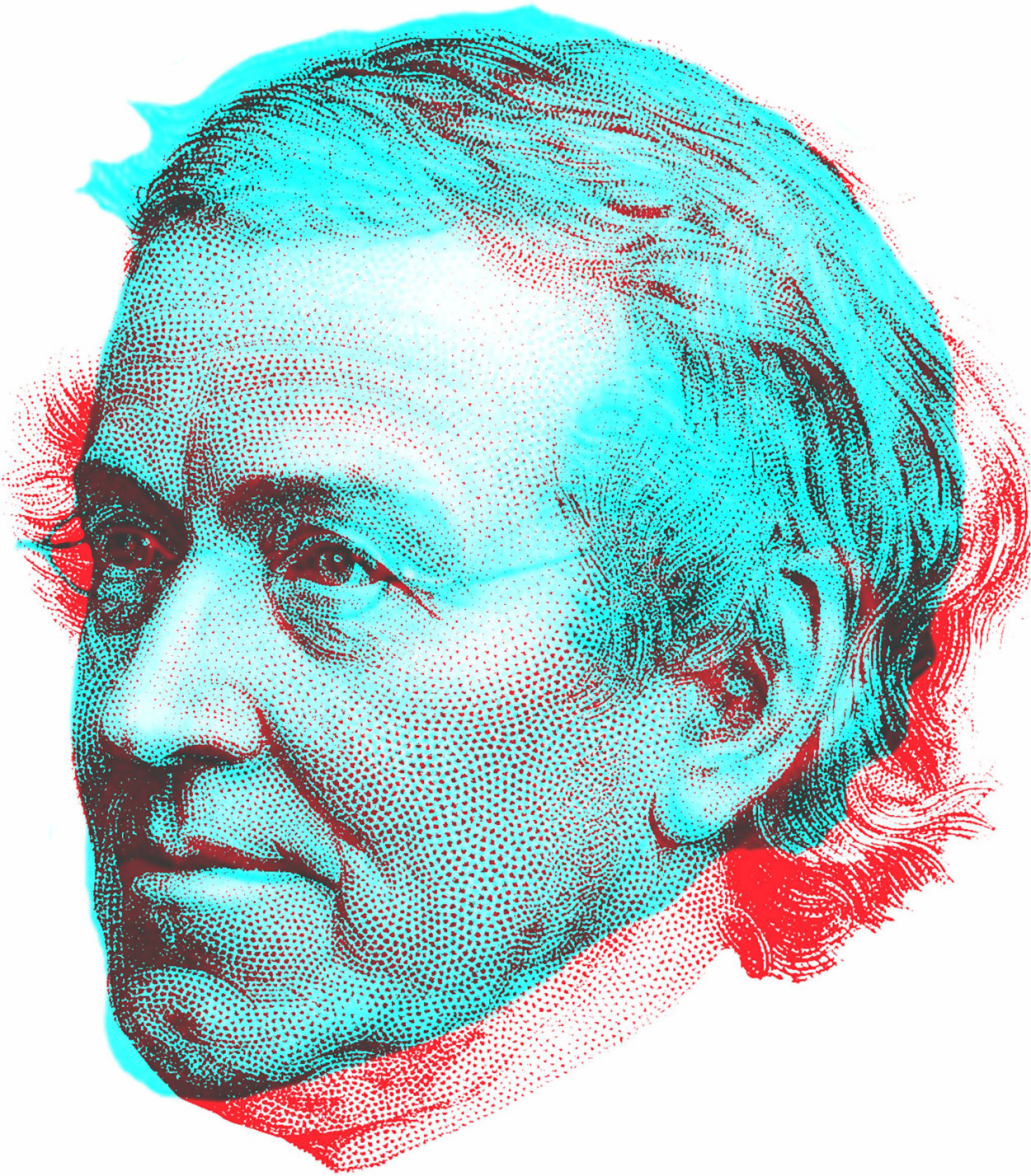
*The ageing Charles Wheatstone* by Nicholas Wade.

Creating rivalry between representations of the same person at different ages depends upon the availability suitable components having similar viewpoints. The general use of three-quarter profile viewpoints in portraiture since the Renaissance is an assistance in this regard. The possibilities increase with the widespread adoption of portrait photography but there are many other options provided by drawings, paintings and engravings in the 19th century. Fortunately, these exist for some of the major contributors to our knowledge of binocular vision, like Wheatstone (Figure 15) and Helmholtz (see Wade, 2021c). The two portraits of Wheatstone are three-quarter profiles derived from engravings.

**Figure 16.**
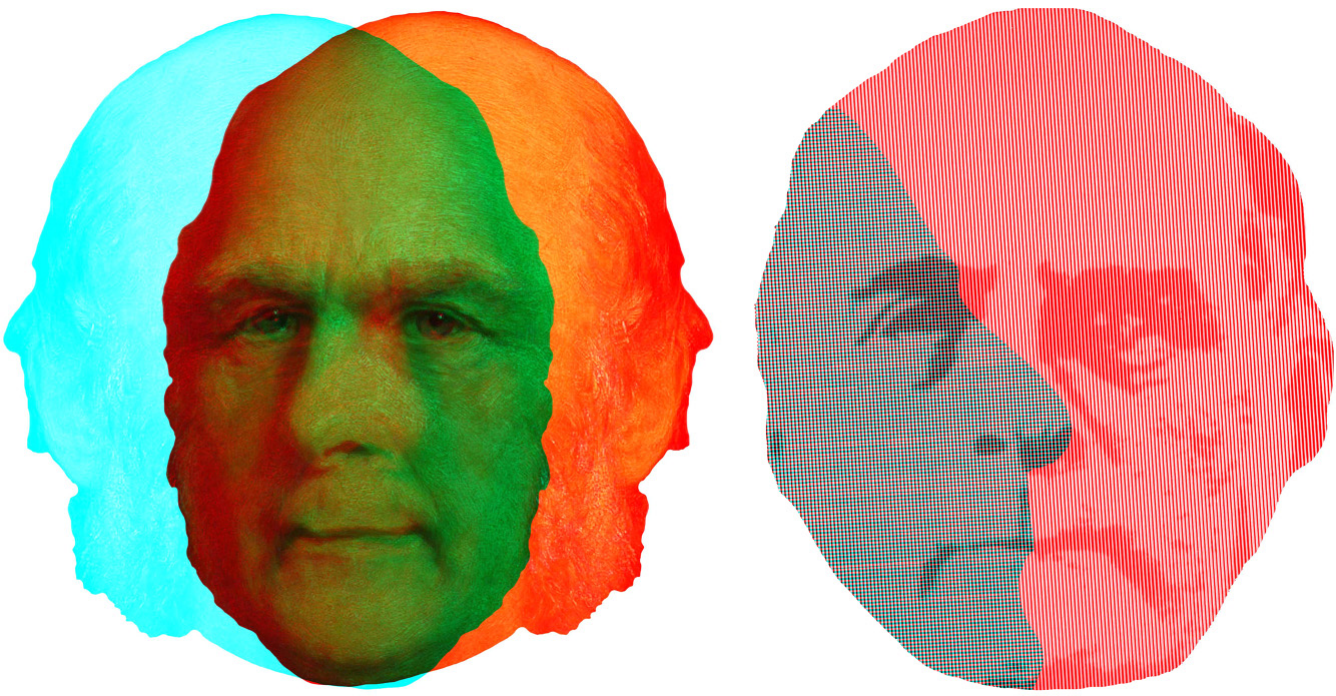
*The faces of Francis Galton* by Nicholas Wade. Left, a portrait of Galton showing combined left and right facing three-quarter profiles with superimposition of the eyes. Right, profile and frontal portraits of Galton; the profile is a silhouette defined by horizontal lines whereas vertical lines define the frontal face. All the facial features are from the frontal face, as can be seen through the cyan filter alone.

Galton (1878) combined portraits of different people in a stereoscope but the purpose was to derive some composite average of human types not to investigate binocular rivalry. Nonetheless, it is clear from Galton's description that rivalry did occur and it was for this reason that composite photographs on a single plate were preferred (see Wade, 2016b). Galton wrote: “Convenient as the stereoscope is, owing to its accessibility, for determining whether any two portraits are suitable in size and attitude to form a good composite, it is nevertheless a makeshift and imperfect way of attaining the required result. It cannot of itself combine two images; it can only place them so that the office of attempting to combine them may be undertaken by the brain. Now the two separate impressions received by the brain through the stereoscope do not seem to me to be relatively constant in their vividness, but sometimes the image seen by the left eye prevails over that seen by the right, and *vice versâ*” (1878, p. 98). Galton was also engaged in using frontal and profile photographs of faces to assist in identifying individuals, particularly criminals, and his proposal was developed by Bertillon (see Wade, 2016b). Both of these aspects of Galton's photographic researches are represented in Figure 16.

From the very beginnings of stereoscopy rivalries have surfaced over priorities, perceptions, and processes. Binocular rivalry can also reflect personal rivalry, and this was certainly the case for Wheatstone and Brewster (see Wade, 1983, 2019, 2023). When Wheatstone published his account of the mirror stereoscope, Brewster's initial reception of it was glowing. However, he later disputed Wheatstone's theoretical interpretation as well as his priority regarding the invention of the stereoscope. They both described investigations of binocular contour rivalry but their interpretations diverged. As was the case for stereoscopic depth perception, Wheatstone argued for central processing whereas Brewster's analysis was peripheral and based on visible direction. They later clashed over Brewster's claim that drawings made by Jacopo Chimenti were made for a 16th century stereoscope (Brooks, 2017; Wade, 2003).

**Figure 17.**
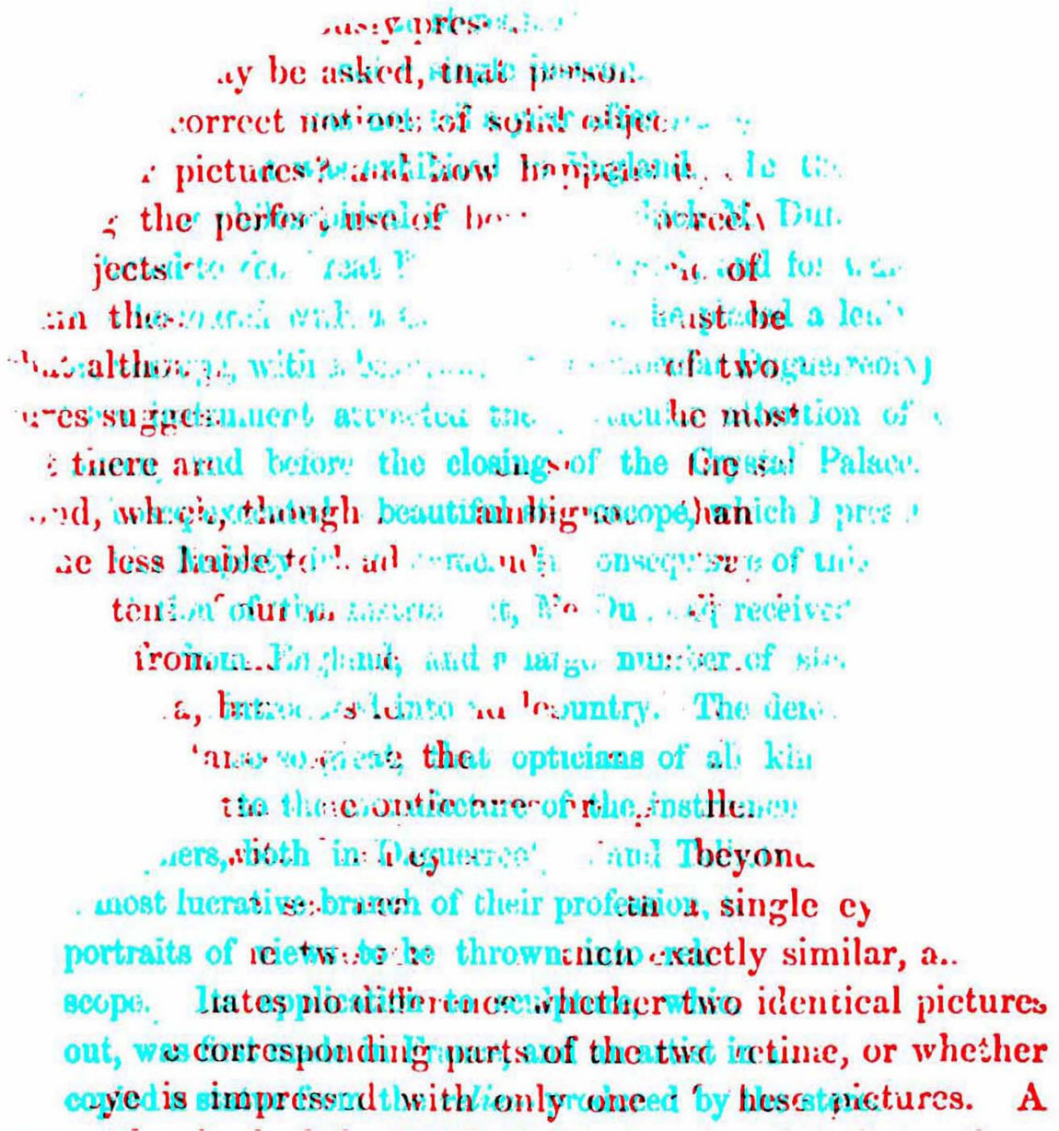
*Binocular rivals—Wheatstone and Brewster* by Nicholas Wade. The dimly defined portraits of Wheatstone and Brewster can be seen in text they wrote describing their stereoscopes.

Many of the disputes between Wheatstone and Brewster were conducted in print and so it is appropriate to show them in textual rivalry (Figure 17). Wheatstone can be seen in a page taken from his article in 1838 in which the stereoscope and experiments with it are described. Brewster is represented in his account from his book on the stereoscope (Brewster, 1856) of the beautiful instrument made by Duboscq that Brewster presented to Queen Victoria at the Great Exhibition held at Crystal Palace in 1851. This account by Brewster himself is the only one available of such a presentation by him and doubt has been cast regarding its actual occurrence (Pellerin & May, 2021).

Both Wheatstone and Brewster investigated binocular rivalry, although it did not have that name at the time of their experiments. Wheatstone (1838) presented a letter A to one eye and a letter S to the other in a stereoscope and noted that they alternated and fragmented. Brewster (1844) observed a similar arrangement of letters and referred to the “capricious disappearance and reappearance of images” (p. 359) as ocular equivocation. Wheatstone remarked that the pattern of perceptual changes was not under voluntary control but he did examine some stimulus factors that could control it, like differences in illumination.

**Figure 18.**
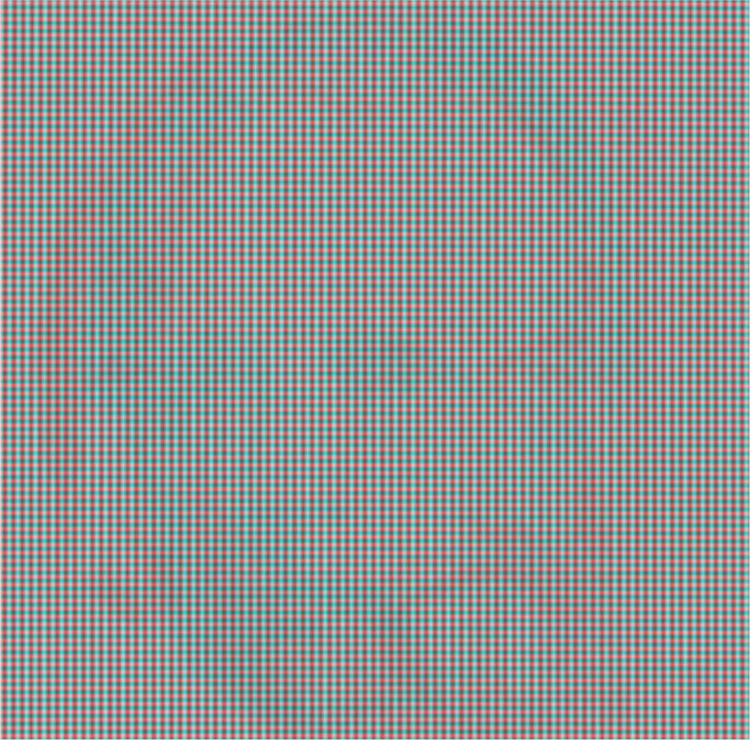
*Binocular rivals—Helmholtz and Hering* by Nicholas Wade. The portrait of Helmholtz is embedded in horizontal lines whereas that of Hering is in vertical lines.

Helmholtz and Ewald Hering (Figure 18) investigated binocular rivalry but, like Wheatstone and Brewster, their interpretations of the phenomenon were also in rivalry (see Turner, 1994; Wade, 2021c). Helmholtz emphasized that stereoscopic depth perception is learned, and that the invention of the stereoscope “made the difficulties and imperfections of the Innate Theory of sight much more obvious than before” (Helmholtz, 1873, p. 274). Thus, the stereoscope gave Helmholtz precisely what he needed to strengthen and defend his own empirical theory of space perception against attacks on it by Hering (see Lenoir, 1993). Helmholtz maintained not only that binocular depth perception is learned but that all spatial perception is founded on judgmental acts based in experience. As Helmholtz saw it, space perception is, from a more general perspective, essentially similar to object recognition. Hering (1861) on the other hand, argued for a physiological interpretation of rivalry. In his *Handbuch*, Helmholtz (1867) discussed rivalry in some detail and emphasized that changing, complex mixtures of the two stimuli tend to be visible most of the time, with only occasional periods in which the stimulus in one eye alone dominates: “This fluctuation, in which parts of the two images mutually supplant each other, either side by side, or one after the other, is what is usually meant by *the rivalry between the visual globes*” (Helmholtz, 1925, p. 494).

**Figure 19.**
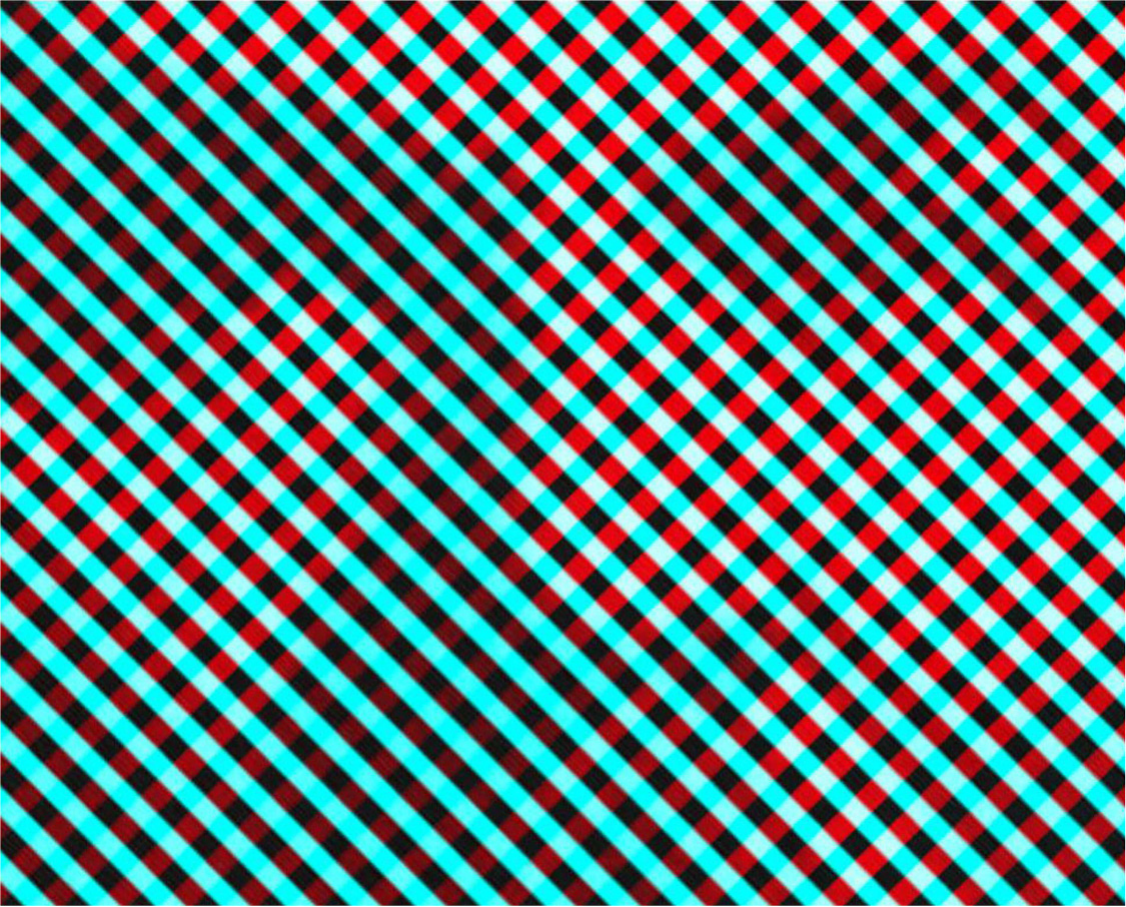
*The rivalry of Burtis Burr Breese* by Nicholas Wade. The portrait of Breese can be seen through the red filter alone.

It was not until the end of the 19th century that some characteristics of rivalry were quantified. Breese (1899) measured the periods of dominance of oblique gratings, like those shown in Figure 19 which also carries his portrait. In addition, Breese described monocular rivalry, which can be experienced with Figures 18 and 19. The fluctuating clarity of one or other grating varies when the pattern is viewed for some seconds with one eye and without the color filters.

**Figure 20.**
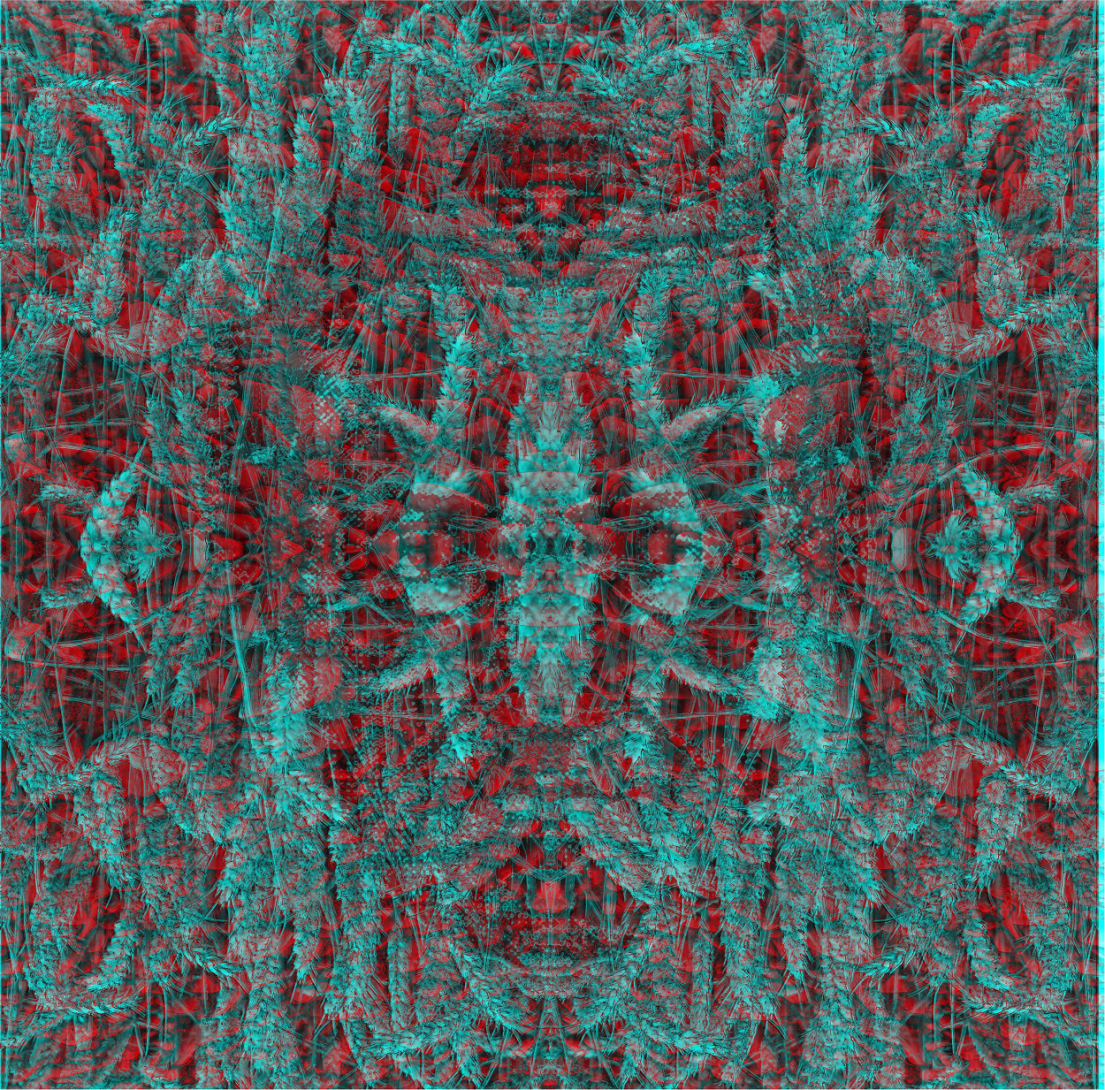
*Wheatstone in rivalry between wheat and stones* by Nicholas Wade. An outline of a frontal portrait of Wheatstone can be seen in the pattern of wheat ears through the cyan filter; a symmetrical pattern of stones is seen through the red filter.

Much more complex patterns than gratings engage in rivalry when presented to different eyes, as can be seen with Figure 20. One eye views a pattern of wheat while the other can see an array of stones; the portrait of Wheatstone is only embedded in wheat!

**Figure 21.**
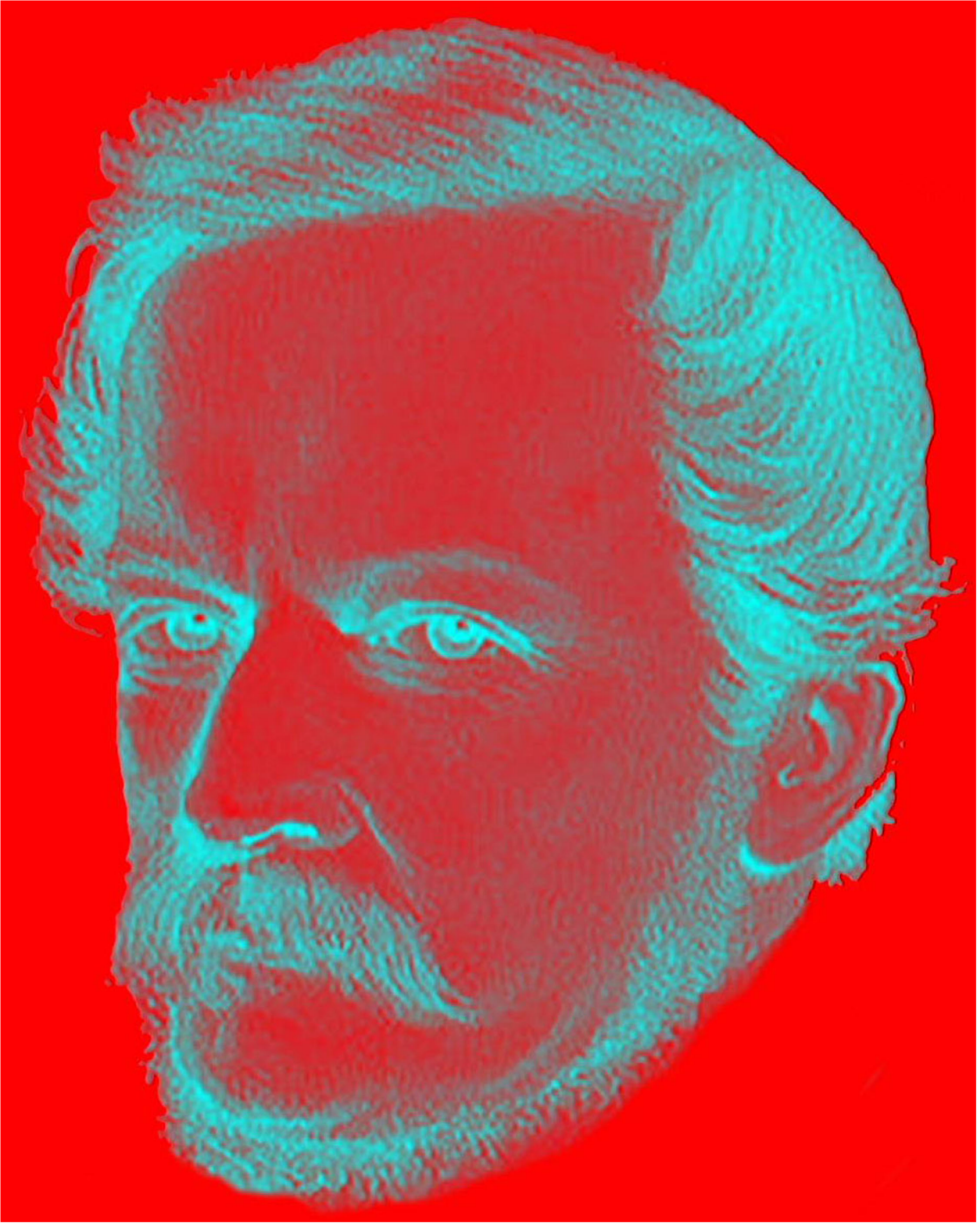
*Lustrous Heinrich Dove* by Nicholas Wade. A positive portrait of Dove can be seen through the red filter and a negative through the cyan. With binocular viewing the skin takes on a metallic sheen.

Rivalry can also be generated by presenting the same image to each eye but a positive to one and its negative to the other. Despite the availability of photography, the initial studies of binocular luster used drawings of geometrical figures either as black on white or white on black. Such combinations were investigated after 1850, first by Dove (1851) followed by Brewster (1861) and others. Dove wrote: “The projection for one eye was drawn in white lines upon a black ground, and for the other eye with black lines upon a white ground. A most remarkable result was obtained by the stereoscopic combination of both. The relief started into existence with surfaces which shone like graphite, having their edges formed of dazzling white and deep black lines which run parallel and in contact with each other throughout” (Dove, 1852, p. 242). Dove referred to it as luster and his portrait (Figure 21) expresses the phenomenon.

**Figure 22.**
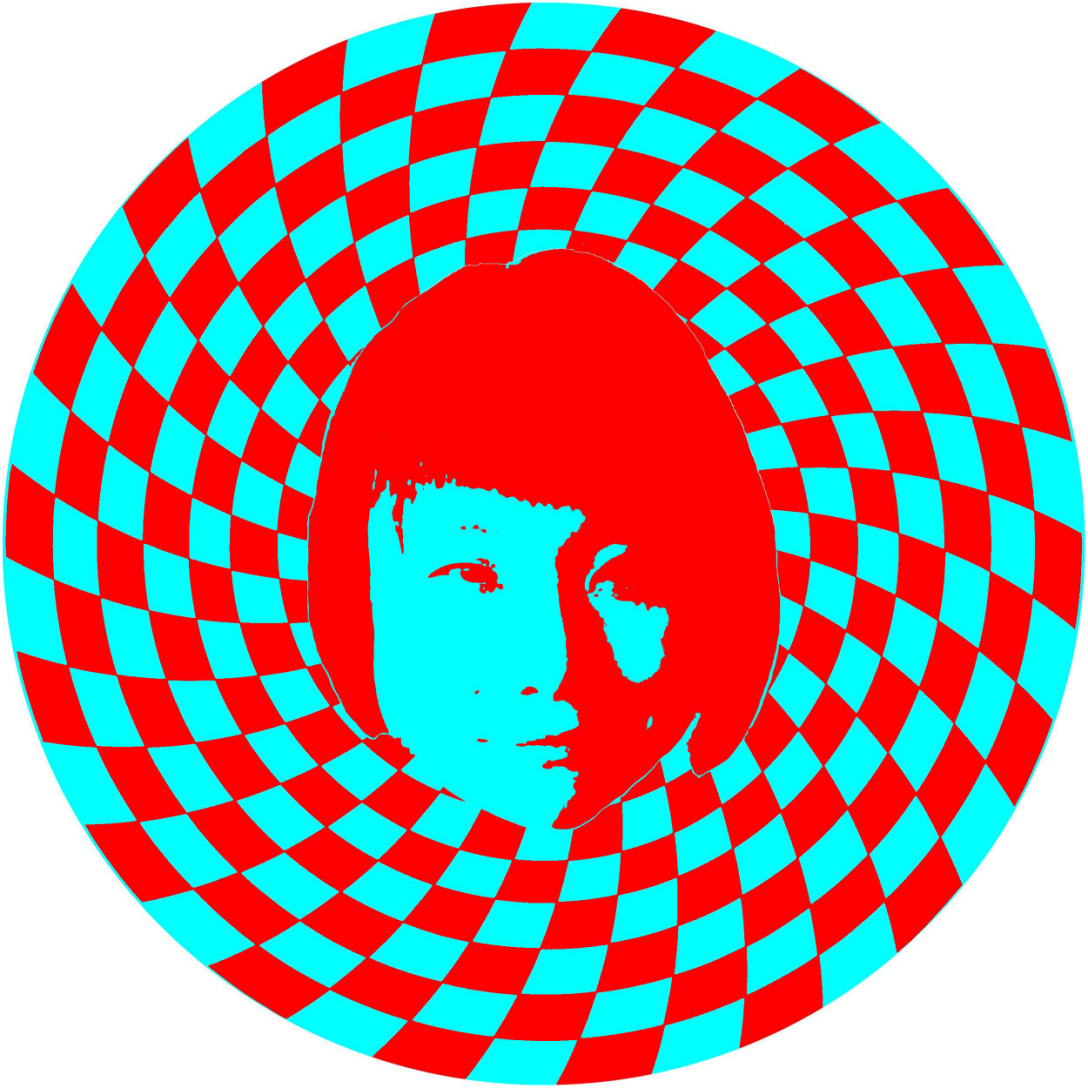
*Yuki Maruyama surrounded by luster* by Nicholas Wade. Maruyama’s portrait is superimposed on a pattern of radiating, curved blocks which appear black in one eye and white in the other when viewed through red/cyan filters. Luster is more pronounced with larger areas without contours as can be seen in the region corresponding to the hair.

One of the few artists explicitly espousing rivalry art is Yuki Maruyama, a Japanese artist whose work is based on binocular luster (Figure 22). She makes large installations painted in red and cyan and provides red/cyan glasses to view them (see https://yukimaruyama.wordpress.com/). The juxtaposition of the large areas of the two colors induces a variety of visual effects and these are enhanced with the lustrous reversals seen when viewing with the red/cyan glasses. Wendt & Faul (2020) have examined the strength of binocular luster with variations in the spatial separations of the contours.

## Portraits in Depth and Rivalry

**Figure 23.**
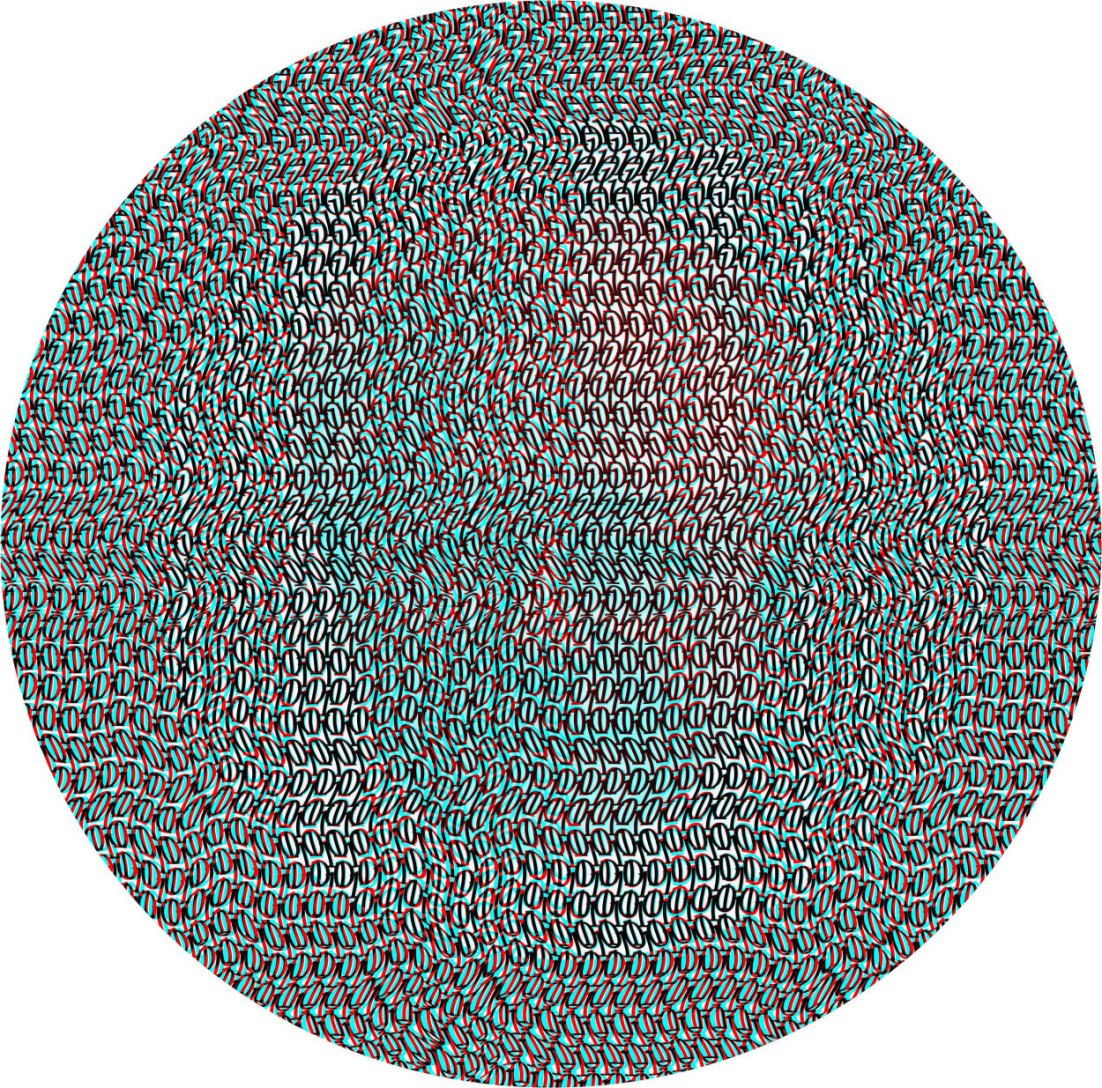
*Béla Julesz in rivalry* by Nicholas Wade. The digital carrier pattern consists of 1s and 0s in a wavy array. A large circle is displaced laterally in one monocular image and the partially transparent portrait of Julesz is embedded in the same monocular image. With binocular viewing the circle in depth remains visible relative to a visually unstable portrait.

Photographic portraits give many signs of their solidity independently of stereoscopic depth. It is for this reason that scientists have tended to use simple patterns like dots and lines in order to investigate depth and rivalry. In the context of rivalry, orthogonal gratings were introduced by Panum (1858) and they continue to be used. Creating images combining rivalry with stereopsis has not been pursued in the context of binocular portraits but it can be (see Wade, 2021d). Figure 23 displays both stereoscopic depth and rivalry using a carrier pattern—a “digital picture” derived from 1s and 0s—rather than computer-generated random dots! Julesz's portrait can be seen in one eye but with two eyes a region in depth is visible as well as evanescent appearances of the portrait. Moreover, the depth remains throughout the periods of visibility and invisibility of the face. The circular region surrounding the portrait reverses in depth with reversal of the filters and it carries the monocular portrait with it.

Compared with conventional random dot stereograms, the carrier patterns used in this and many other binocular portraits are not as geometrically regular. Indeed, some degree of irregularity is common. It can be a spatial constituent of the pattern, as is the case for naturally occurring textures, or it can be graphically introduced, as in Figure 23. Regular arrays of 1s and 0s were distorted and superimposed so that the displacement of a region (here a large circle) in one monocular image cannot be detected until it is combined binocularly with its untransformed partner.

**Figure 24.**
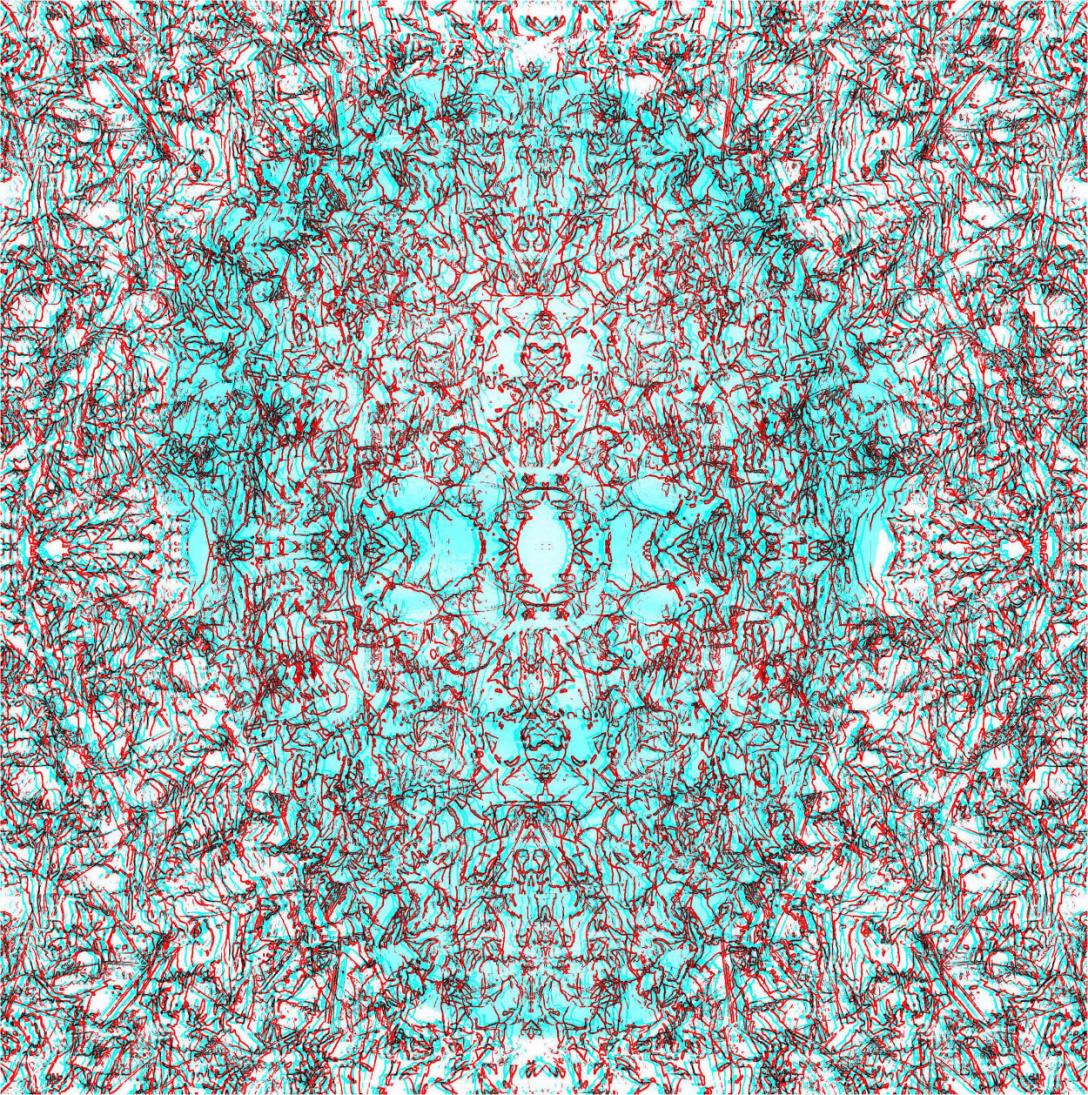
*The rivalry of Pim Levelt* by Nicholas Wade. The carrier pattern is based on a photograph of ivy leaves that was multiplied, graphically manipulated and symmetrically superimposed. The partially transparent portrait is present in one monocular image and the four concentric annuli are displaced in the other.

In the first half of the 20th century there was little experimental research on binocular rivalry relative to that on stereoscopic vision. At around the time that Julesz (1960) demonstrated stereopsis with random dot patterns, increased interest was again directed to rivalry (see Blake, 2005, 2017; Wade, 2021d). For example, Levelt (1965) conducted detailed examinations and quantitative analyses of rivalry and hastened the application of models to account for the patterns of rivalry fluctuations. Among the stimuli he employed were rings and discs, rather like the annuli that can be seen in depth in Figure 24; Levelt's portrait is presented to one eye only. Unlike the portrait in Figure 23, the concentric rings in depth overlap the face so that it is not seen in the same depth plane and this applies for both arrangements of the color filters.

**Figure 25.**
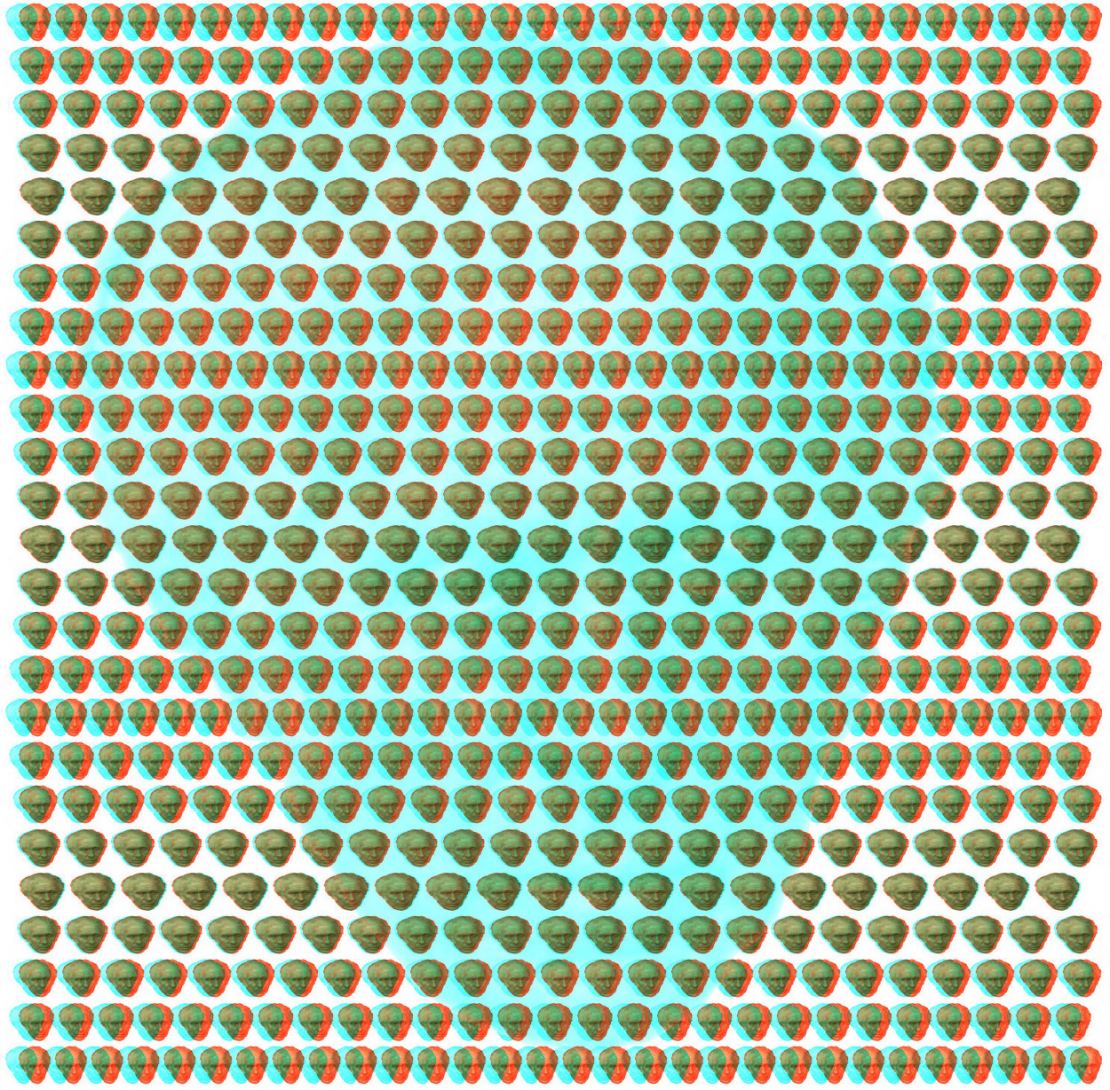
*Brewster in rivalry and autostereoscopic depth* by Nicholas Wade. The rows of small portraits of Brewster vary in size systematically so that they appear as peaks and troughs. The large portrait of Brewster (presented to one eye alone) is best seen when the pattern is viewed from a distance.

A binocular depth phenomenon was described before Wheatstone invented his stereoscope but it is dependent on convergence rather than retinal disparity. It was initially described early in the 19th century in the fluted marble of a mantelpiece (Blagden, 1813). With under-convergence so that adjacent elements were fused the fluting appeared to be further away and magnified relative to fixating on the same elements. However, its significance was not appreciated until after the invention of the stereoscope. Brewster (1844) rediscovered the illusion when observing a repetitive pattern of flowers printed on wallpaper. It was from such patterns that were frequently so printed that the phenomenon derived the name “wallpaper illusion” (see Wade, 2016a). Repetitive patterns on wallpaper can be seen to lie in front of or behind the plane of the wall if adjacent elements are fixated. Small and systematic variations of the size and spacing between the elements can introduce relative, stereoscopic depth. Slight variations in the locations of the repetitions along rows can be introduced to add stereoscopic depth, as is shown in Figure 25. It consists of an array of portraits of Brewster which results in the surface no longer looking flat but stepped in depth from top to bottom; the apparent depth reverses with reversal of the color filters. Depth can be seen in the pattern without the color filters by over-converging or under-converging to fuse adjacent portraits. When variations in depth are apparent in patterns like this they are called autostereograms. It works best with combining adjacent portraits in the rows of the largest heads. The depth can be seen more easily with the aid of the color filters. Both the large monocular portrait of Brewster and those forming the array are from a photograph taken by his friend John Adamson in 1855 (see Morrison-Low and Bruce, 2018).

## Artists Employing Stereoscopy

**Figure 26.**
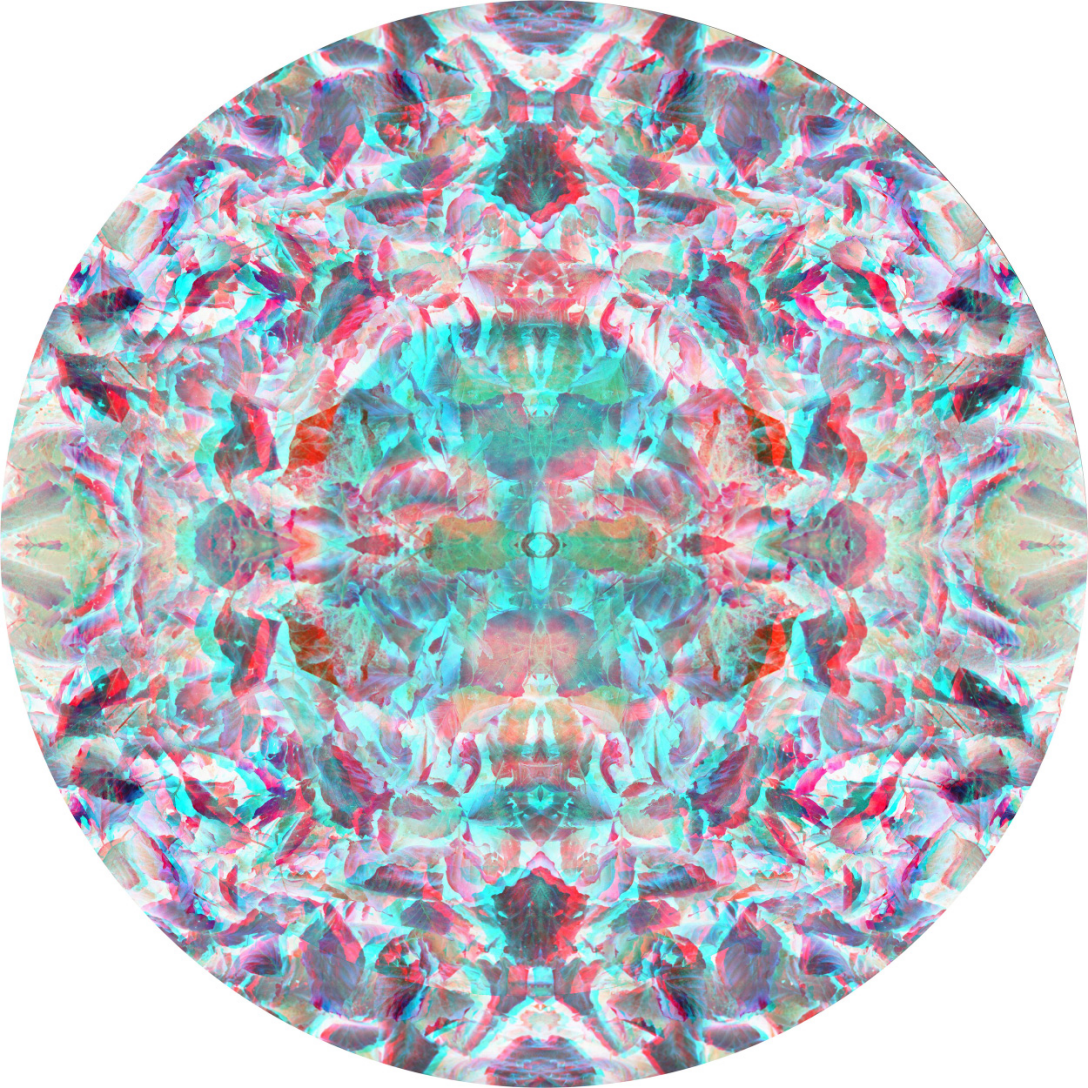
*Surreal Salvador Dali* by Nicholas Wade. The carrier pattern is derived from a photograph of fallen autumn leaves and the portrait shows a mustachioed Dali surrounded by a circle in depth. The final image is a negative of the anaglyph.

Some artists have produced binocular pictures expressing both stereoscopic depth as well as binocular rivalry though few have combined the two. The difficulties involved in introducing small and systematic disparities in paired pictures was appreciated by Wheatstone (1838, 1852) who suggested a solution to it: “At the date of the publication of my experiments on binocular vision, the brilliant photographic discoveries of Talbot, Niepce and Daguerre, had not been announced to the world. To illustrate the phenomena of the stereoscope I could therefore, at that time, only employ drawings made by the hands of an artist. Mere outline figures, or even shaded perspective drawings of simple objects, do not present much difficulty; but it is evidently impossible for the most accurate and accomplished artist to delineate, by the sole aid of his eye, the two projections necessary to form the stereoscopic relief of objects as they exist in nature with their delicate differences of outline, light and shade. What the hand of the artist was unable to accomplish, the chemical action of light, directed by the camera, has enabled us to effect” (1852, pp. 6–7). Nonetheless, some artists have painted paired portraits and Salvador Dali is the most prominent among them (Ades, 2000, 2008). Most of Dali's stereoscopic paintings were produced in the 1970s (see King, 2018). He made many stereoscopic works and also interacted with Julesz (see Julesz, 1995). A surreal Dali can be seen in rivalry and surrounded by depth in Figure 26.

**Figure 27.**
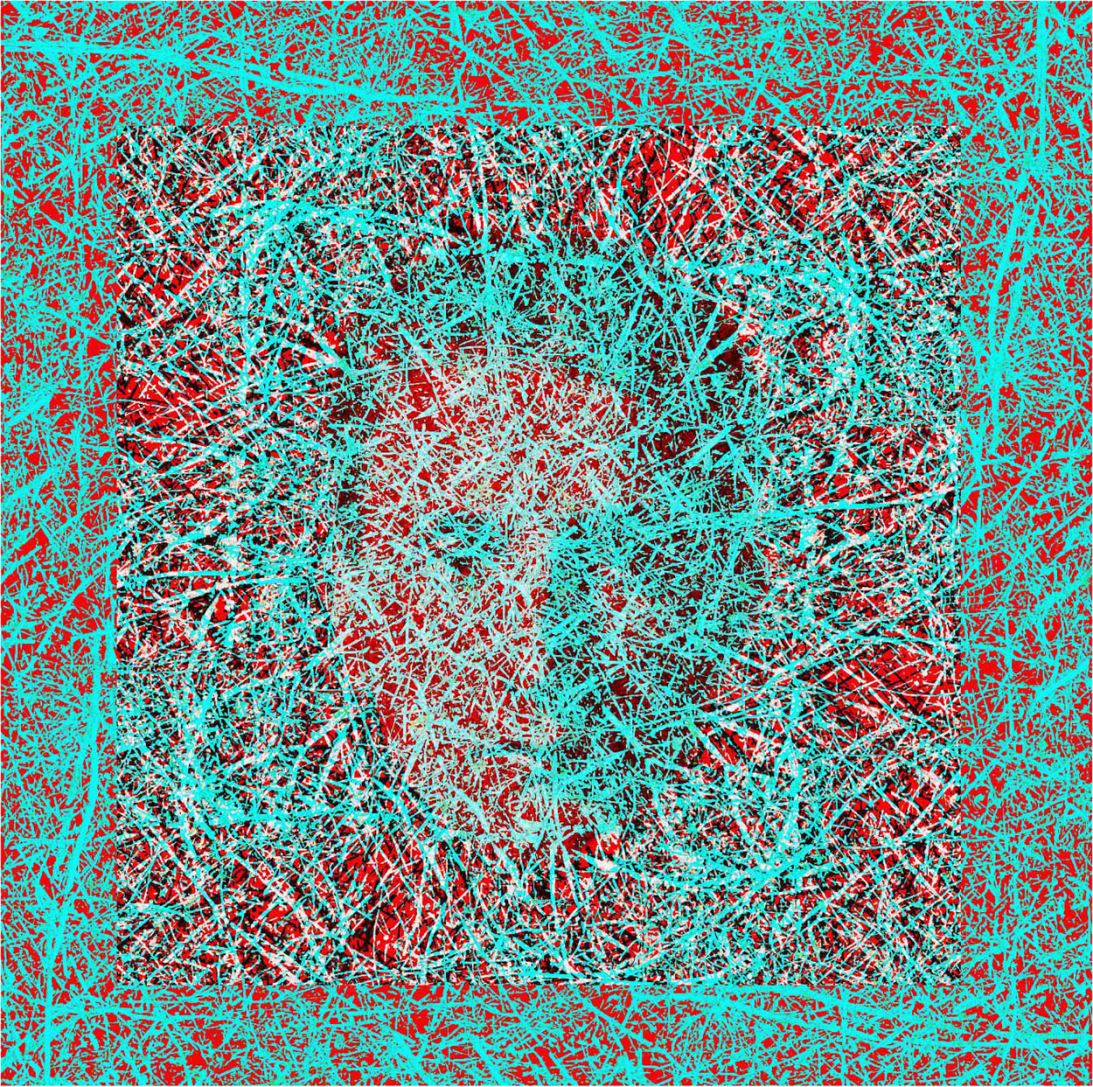
*Marcel Duchamp in rivalry and depth* by Nicholas Wade. The portrait of Duchamp can be seen through the red filter and the carrier pattern for the surrounding square in depth is derived from a photograph of tree branches.

Both Dali and Marcel Duchamp made works in which the left and right eye images were radically different and engaged in binocular rivalry (Ades, 2008; King, 2018). Duchamp's stereoscopic works were produced much earlier than Dali's and tended to be modifications of stereoscopic photographs. Duchamp is represented in rivalry in Figure 27; the carrier pattern has the appearance of scratchings on film and these marks make the square frame in depth surrounding the portrait more difficult to discern. The attraction of stereoscopic works for both Dali and Duchamp was to amplify the engagement of the viewers with the works before them.

**Figure 28.**
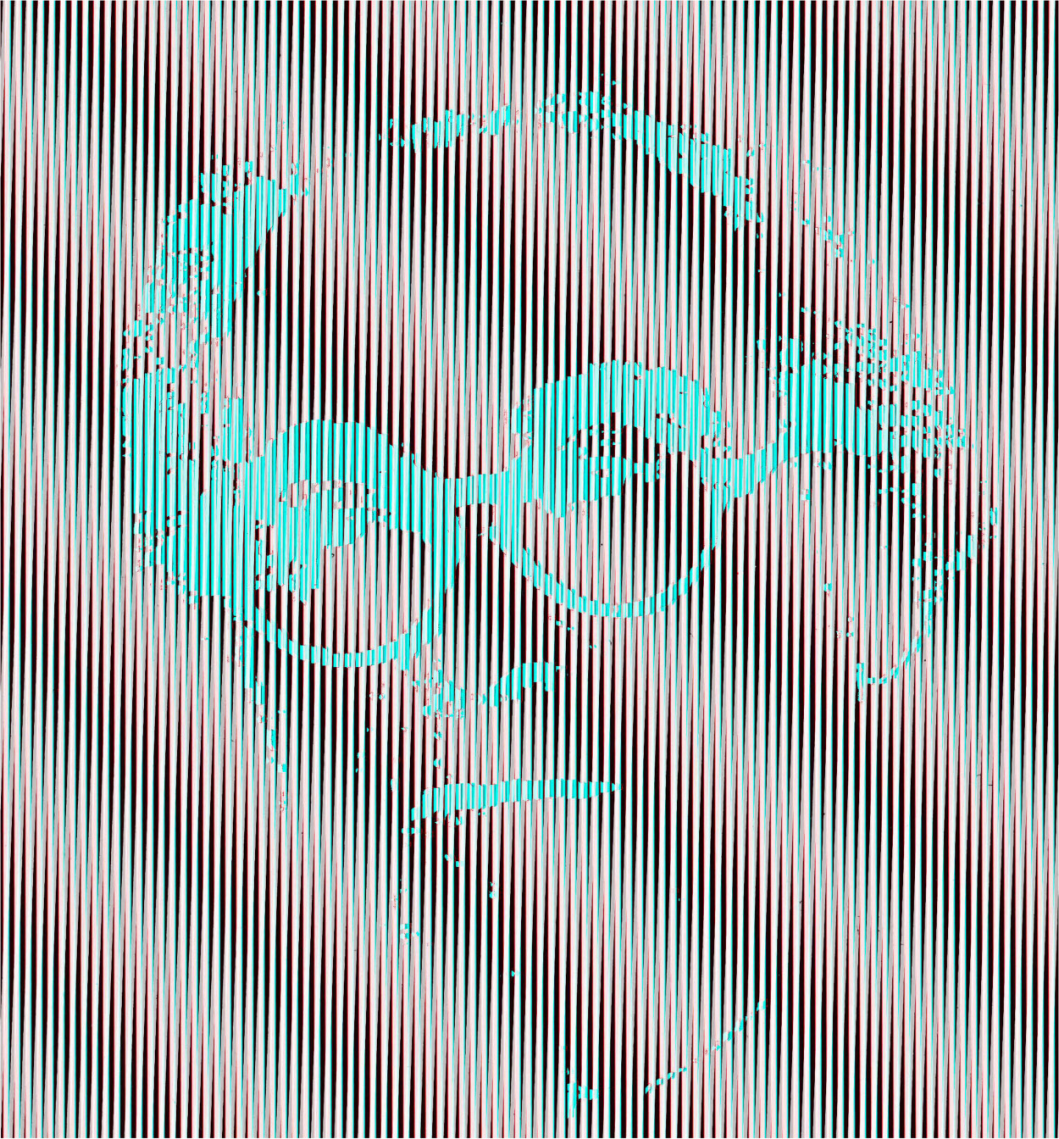
*Ludwig Wilding in moiré fringes* by Nicholas Wade. Both monocular images contain a portrait of Wilding embedded in a grating but one is positive and the other is negative. The inclined moiré fringes are produced by an inclination of one monocular grating with respect to the other.

A stereoscopic technique involving repetitive patterns was introduced in an artistic context by Ludwig Wilding (1977, 2007). It is based on disparities between moiré fringes generated by the interference of regular repetitive patterns (like gratings) separated slightly in depth. Moreover, the apparent stereoscopic space varies with the viewing distance of the observer (because disparity between the moiré fringes varies with viewing distance). The depth can be produced from curved as well as flat surfaces, and opposite directions of depth are often incorporated in the same work. The relationship between the spatial frequencies of the transparent and printed patterns determines the direction and amount of the depth seen, and they can be given precise mathematical descriptions (Kondo et al., 1990). Depth can be seen as a consequence of the disparities of the moiré fringes when the head is stationary, and it can be augmented by lateral head movements which yield motion parallax between the moiré patterns. It is difficult to do justice to the moiré effects generated by physical separations in depth on a flat surface and so Wilding is represented in moirés from inclined gratings (Figure 28).

**Figure 29.**
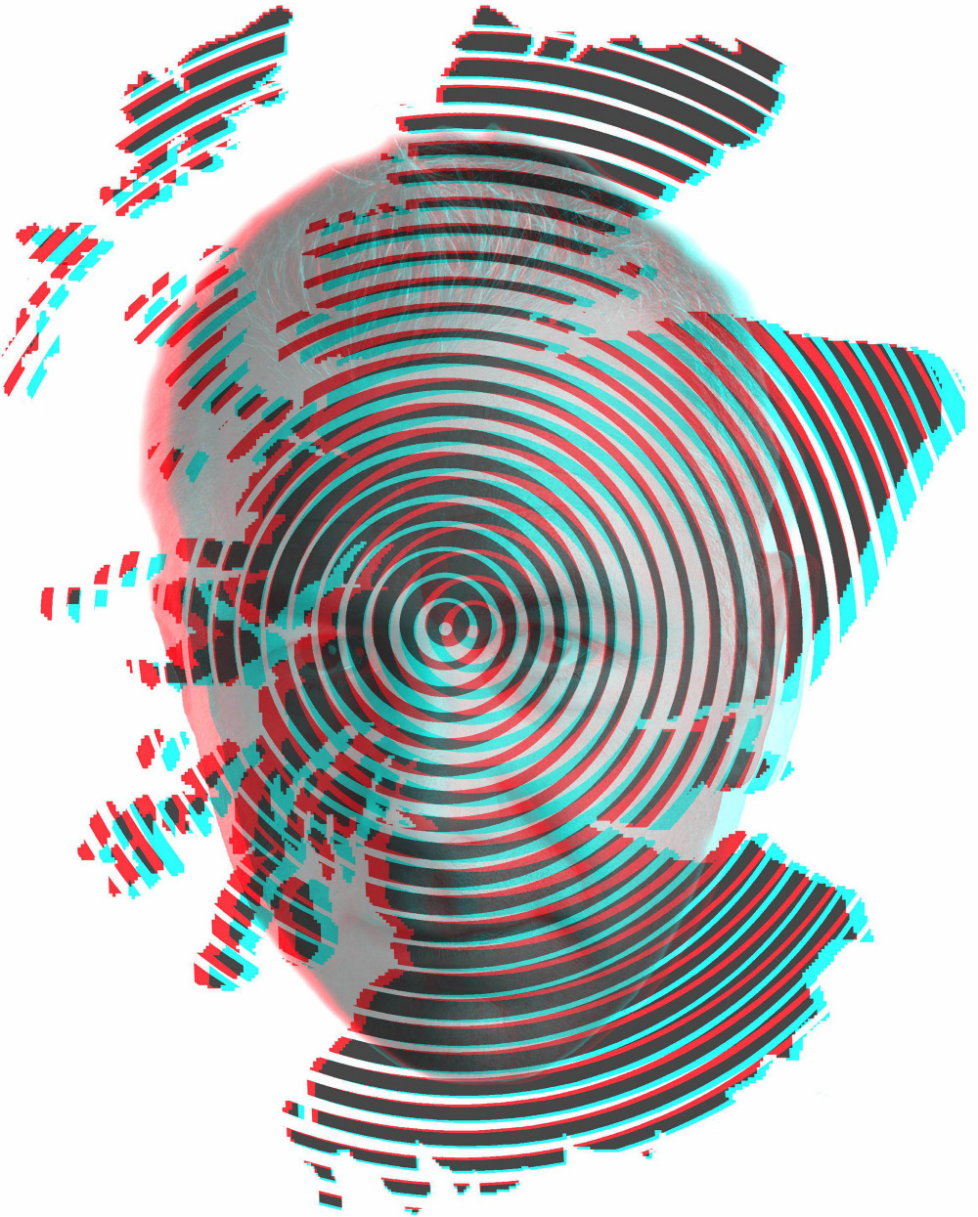
*Calum Colvin in Scotland* by Nicholas Wade. A portrait of Colvin in disparity is combined with a stylized map of Scotland defined by eccentric circles. The central point of the design is aligned with the cyclopean eye and the peak of the highest mountain in Scotland.

Calum Colvin (Figure 29) commenced his artistic endeavors by painting scenes over three-dimensional objects with a view from a particular station point. Alignment was maintained so that the final photographic image was taken from the only point from which this was possible. By adopting two viewpoints, neither of which will yield perfect alignment between the contours painted on the solid objects, retinal disparity can be introduced. The clues to the objects are given visually rather than conceptually. Even so, disparity takes time to develop and our familiarity with pictorial images tends determine the initial visual victory. Some of his stereoscopic works have been portraits of Wheatstone and Brewster (Colvin, 2009; Wade, 2009). Depth derived from disparity is in competition with pictorial depth, so that the art is not stereoscopic in the narrow sense but the works display a dynamic duel between the pictorial and binocular cues to depth. In one sense the binocular works of Colvin revert to the approach adopted by Wheatstone and Brewster, but in another they add a delightful twist to this stereoscopic tale. By incorporating rivalry with stereoscopy he has extending the art of the third dimension.

**Figure 30.**
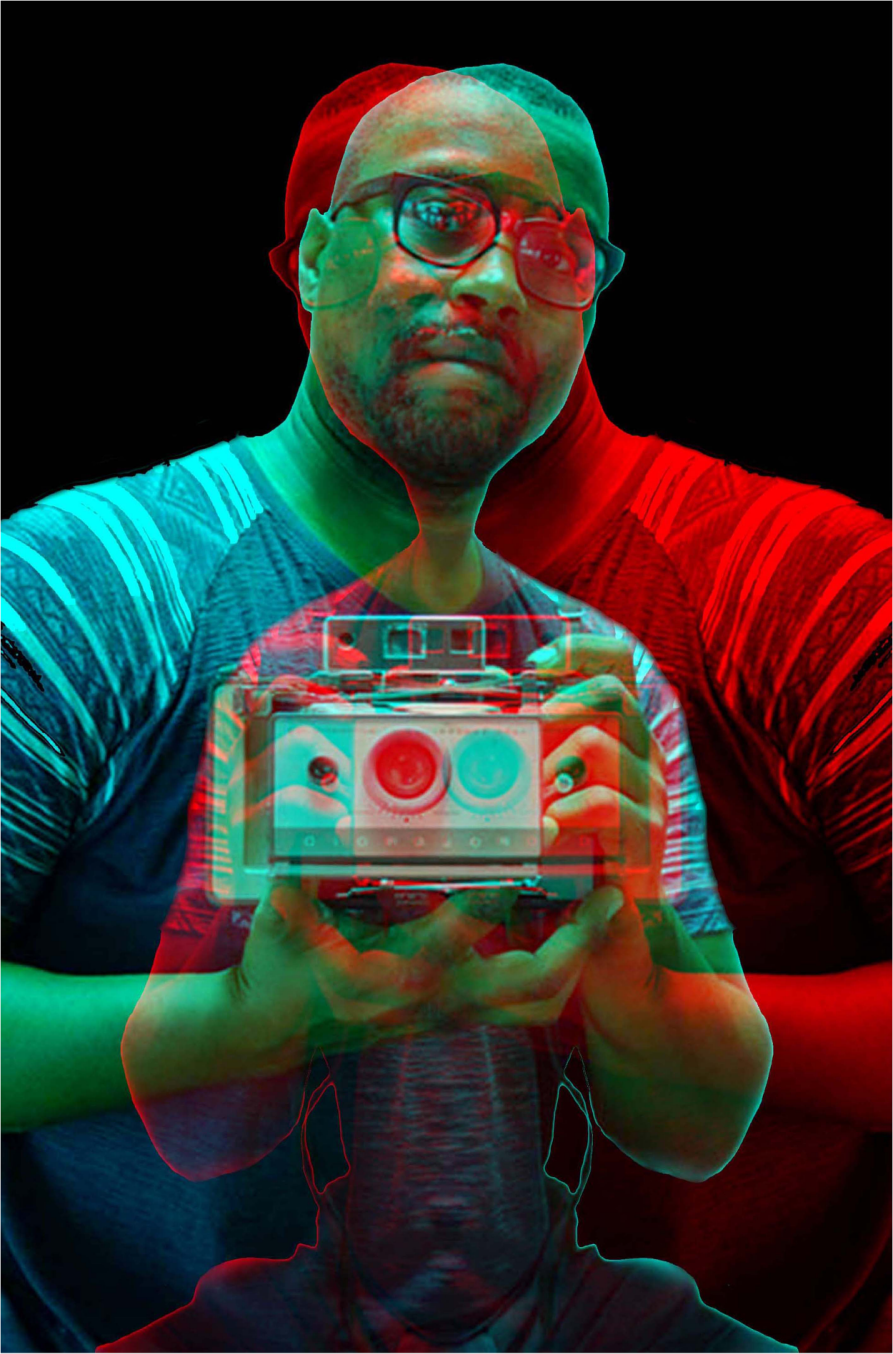
*Antonio McAffee in rivalry* by Nicholas Wade. The central eyes of the left/right reversed portraits of McAffee are aligned which results in the disparate locations of the lens in the hand-held camera. There is a strong tendency to fuse the lenses of the disparate cameras resulting in the impression of its depth and enhanced rivalry of the background.

The artist Antonio McAfee (Figure 30) enlists anaglyphic images in his examinations of historical photographs of middle-class African Americans. He reprocesses old portraits often combining them with their left–right reversals to produce symmetrical anaglyphs which are viewed with red/cyan glasses (see https://antoniomcafee.net/). The anaglyphic portraits can be partially masked with yet other images superimposed on them. These techniques have been used to make the anaglyphic portrait of McAfee; the reflected images are centered on a common eye and the portraits are masked in black.

**Figure 31.**
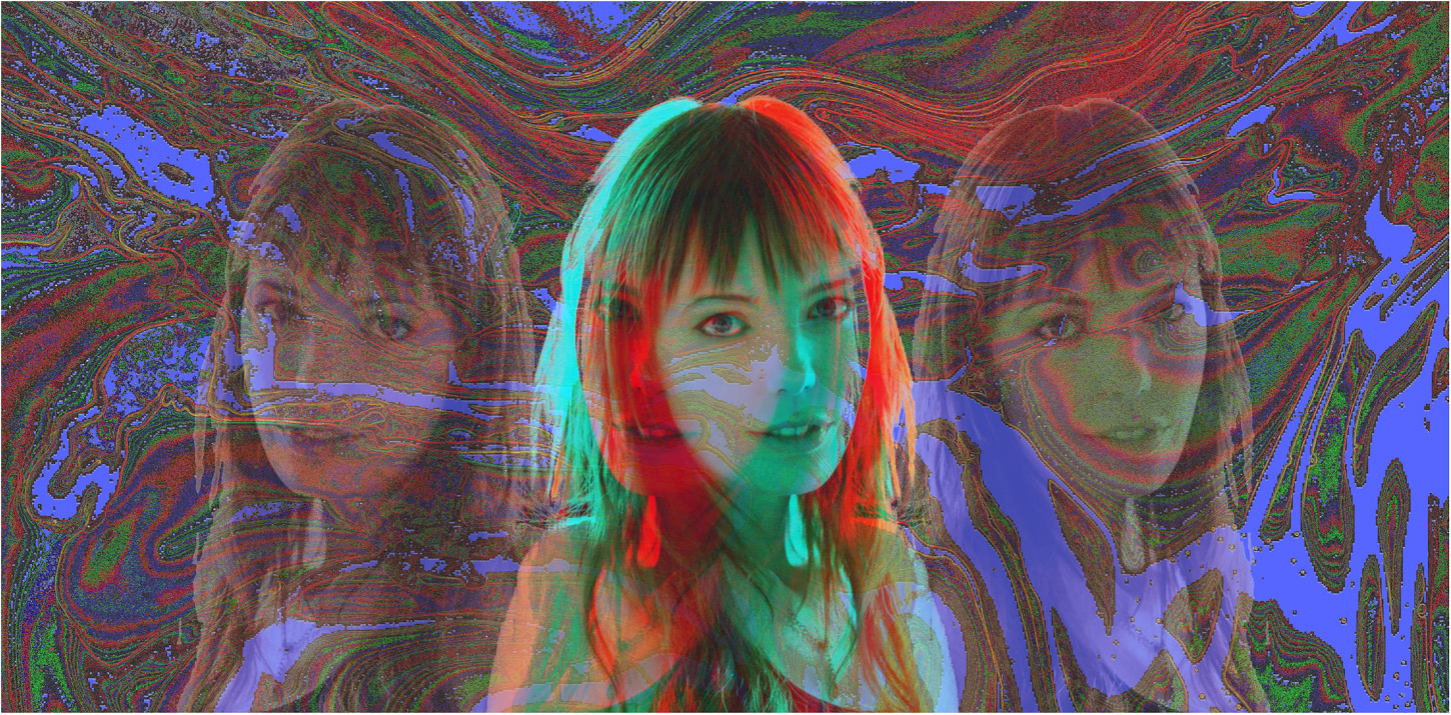
*Surreal Sara Le Roy* by Nicholas Wade. The flowing background pattern partially obscures the lateral portraits but not the central ones which are left/right reversals centered on an eye.

Stereoscopic art is undergoing a resurgence of interest in artistic circles but not in terms of the conventional representation of depth from disparity. An example of this is the work of Sara Le Roy who combines narrative painting with stereoscopic imagery (see https://saraleroy.com/selected-art-works). Much of her work interweaves dark and surreal themes with anaglyphic depth so that the stereoscopic effects are but a part of a partially painted scene. The paintings are made up of layers, starting from the most distant and photographs are taken of each layer. The photographs are assembled to produce anaglyphs and then printed on canvas which is painted yet again. Sara Le Roy is shown in anaglyphic combination with conventional portraits embedded in a surreal background (Figure 31). The background is derived from a flowing abstract painting that was photographed and digitally manipulated.

**Figure 32.**
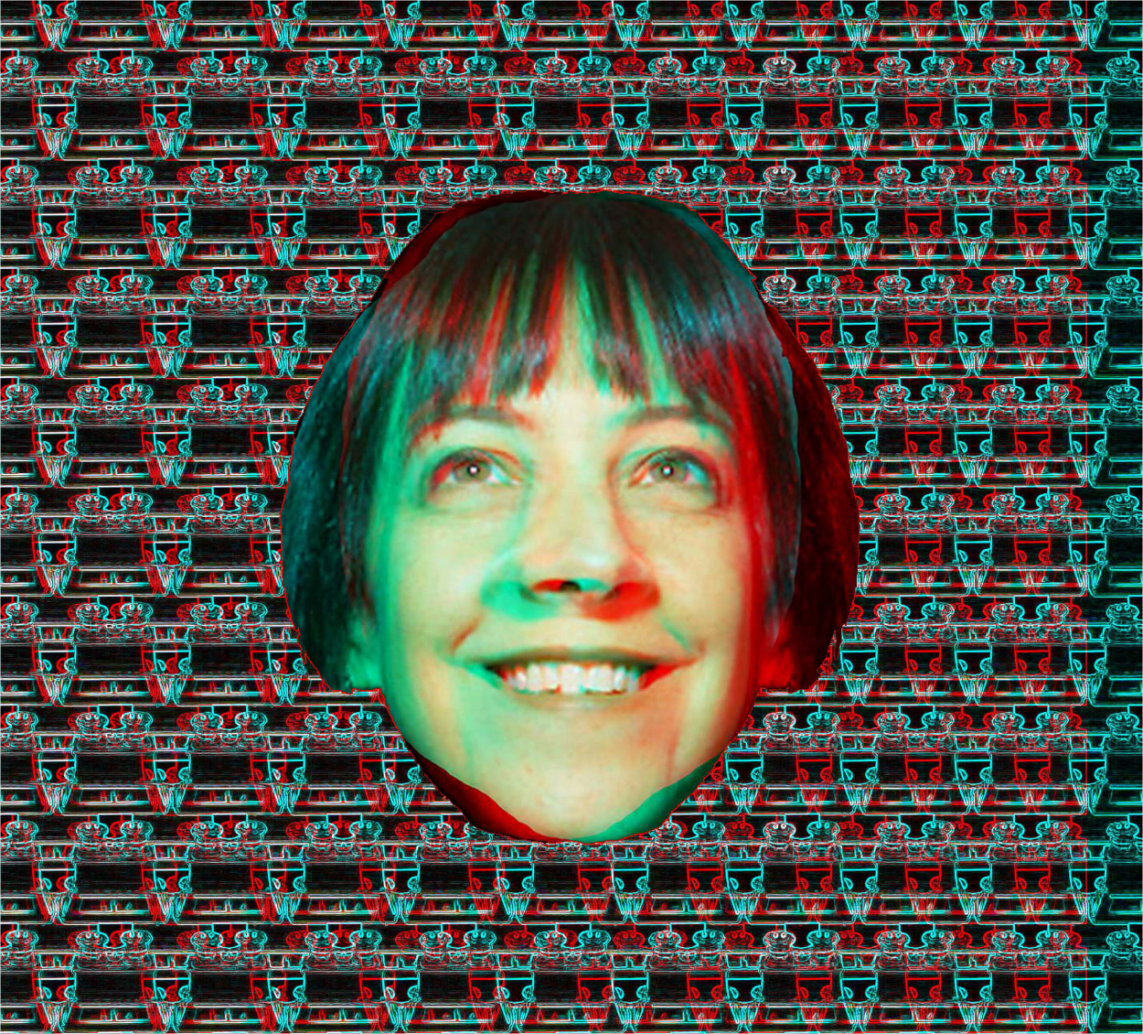
*Twin portraits of Colleen Woolpert* by Nicholas Wade. The symmetrical portraits of Woolpert are superimposed on an array of stereoscopic stereoscopes the right side of which either approaches or recedes depending on the arrangement of the red/cyan filters. The portrait appears to rotate with the background.

Colleen Woolpert is a photographer whose stereoscopic works conflate the twin pictures to be viewed with those of herself and her twin sister. While they share genes they do not see the world in the same way. Colleen's sister does not see stereoscopic depth, due to childhood strabismus, and that has been a stimulus for producing stereoscopic photographs (see https://stereoscopy.blog/2022/06/21/the-art-of-stereoscopy-colleen-woolpert/). Moreover, Woolpert has produced particular models of stereoscopes for viewing paired pictures which she calls TwinScopes (see https://colleenwoolpert.com/TwinScope-Artist-Talk). A double portrait of her is shown in Figure 32 together with a background of stereoscopic Brewster stereoscopes, not unlike Woolpert's TwinScopes. Initial interest is concentrated on the unstable, overlapping faces but longer observation reveals that the pattern of stereoscopes starts to rotate on the right side, either toward or away from the viewer (depending on the arrangement of eyes and filters). As the right hand side of the background recedes it appears to grow whereas when it approaches it shrinks so that the frame no longer looks rectangular.

Sebastian Cramer (2022) takes stereoscopic photographs of plants and prints them as large anaglyphs. He also makes anaglyphic portraits of forward facing heads against white backgrounds (https://sebastiancramer.com/pages/portraits). The exquisite detail in the anaglyphs can be seen with or without red/cyan filters and the disparities tend to be confined to hair rather than the frontal features of the face. That is, the stereoscopic effects are minimal but this does not detract from the power of the portraits. In one sense, Cramer's anaglyphic portraits speak to the issues raised here—without surrounding objects there are relatively small disparities within the face and the binocular portraits are not greatly different from those seen by one eye alone. This is one of the reasons why a different approach has been adopted here for engaging binocular processes of cooperation and competition in creating portraits for two eyes.

## Conclusions

Binocular portraits, like binocular art in general, can be characterized as revealing to two eyes what is concealed from one. That is, processes of stereopsis and rivalry as well combinations of them are expressed in the representations of individuals. Binocular portraits have traditionally reflected two views of the same person photographed from laterally different positions. Unless the lateral separations are large there will only be small differences between the photographic images of a face viewed by one or two eyes. The binocular portraits illustrated in this article draw upon the same interactive processes involved in all vision with two eyes but they are expressed in different ways. In most cases the binocular portraits are derived from single paintings, engravings or photographs of pioneers and practitioners of binocular vision. Manipulations have been made of disparate backgrounds in order to induce depth or rivalry in the singular images. In the past, rivalry has been neglected as an artistic aid relative to stereopsis. An attempt is made here to shift the balance a little toward binocular portraits involving rivalry either alone or in combination with stereopsis.
